# Assessing methods to identify Veterans Health Administration populations with Veterans Justice Programs contact to improve health research

**DOI:** 10.1371/journal.pone.0357665

**Published:** 2026-09-08

**Authors:** Andrea K. Finlay, Ingrid Binswanger, Alex H.S. Harris, Matthew Stimmel, Mengfei Yu, Kreeti Singh, Jack Tsai

**Affiliations:** 1 Center for Innovation to Implementation, VA Palo Alto Health Care System, Palo Alto, California, United States of America; 2 VA National Center on Homelessness Among Veterans, Veterans Health Administration, Washington, District of Columbia, United States of America; 3 Division of Health Systems Science, Department of Medicine, UMass Chan Medical School, Worcester, Massachusetts, United States of America; 4 Institute for Health Research, Kaiser Permanente Colorado, Aurora, Colorado, United States of America; 5 Colorado Permanente Medical Group, Denver, Colorado, United States of America; 6 Division of General Internal Medicine, University of Colorado School of Medicine, Aurora, Colorado, United States of America; 7 Department of Health Systems Science, Bernard J. Tyson Kaiser Permanente School of Medicine, Pasadena, California, United States of America; 8 Stanford-Surgery Policy Improvement Research and Education Center, Department of Surgery, Stanford University, Stanford, California, United States of America; 9 Veterans Justice Programs, Department of Veterans Affairs, Washington, District of Columbia, United States of America; 10 School of Public Health, University of Texas Health Science Center at Houston, Houston, Texas, United States of America; 11 Department of Psychiatry, Yale University School of Medicine, New Haven, Connecticut, United States of America; University of Foggia: Universita degli Studi di Foggia, ITALY

## Abstract

Military veterans with criminal legal involvement have a need for healthcare and housing to attenuate their risks of suicide, overdose, and homelessness. Links between criminal legal involvement and Veterans Health Administration (VHA) care are poorly understood, partly because it is challenging to correctly identify veterans with criminal legal involvement. This study’s primary objective was to assess VHA data sources and codes from the electronic health record that indicate contact with Veterans Justice Programs (VJP) staff – a broad proxy for criminal legal involvement. We examined three data sources used in previous research to identify veterans who had contact with VJP staff: (1) presence of VJP clinic stop codes in the outpatient encounter records, (2) Homeless Outreach Management and Evaluation System (HOMES) VJP form records collected during VJP outreach, and (3) chart note titles that included VJP terms. In Fiscal Year 2024, there were 52,672 unique VHA-eligible veterans with VJP contact as determined by at least one indicator: 80% with clinic stop codes, 22% with HOMES records, and 63% with chart note titles (Fleiss’ κ = 0.22, *p* < .001). Patient characteristics varied in their association with each data source, suggesting substantial proportions of veterans with specific characteristics who received VJP services would be missing when only one or two data sources were used. Depending on the data source, diagnosis of a clinical condition varied by 9% and there was a 6% difference observed in a performance measure. These results suggest that the use of all three data sources to identify veterans with VJP contact is the most comprehensive strategy among those examined. Results are most applicable to VHA, the largest healthcare system to serve a criminal legal involved population, and provide information that other healthcare systems may need various data sources and indicators to identify patients with criminal legal involvement.

## Introduction

Military veterans constitute a small percentage of people involved in the criminal legal system, representing about 8% of the prison incarcerated population in the United States in 2016 [1]. Other forms of criminal legal involvement experienced by veterans include being arrested or involved in court proceedings, incarcerated in jails, and under probation or parole [2]. Veterans with criminal legal involvement are a high priority population at the Veterans Health Administration (VHA) because of their elevated risks of suicide attempts and death, overdose death, and homelessness [3–5], which are top clinical priorities for the VHA. They also have higher rates of mental health and substance use disorders and some medical conditions, including bronchitis/chronic obstructive pulmonary disorder, hepatitis B or C, or HIV, compared to veterans without criminal legal involvement, and they may struggle to find housing and employment [6–10]. Ensuring these veterans receive needed healthcare and housing resources is important to improve their health and quality of life.

To assist veterans with criminal legal involvement and link them to needed healthcare, housing, and other indicated services, the VHA created the Veterans Justice Programs (VJP) which is housed under the Homeless Programs Office [11]. The two VJP programs are Health Care for Re-entry Veterans (HCRV), which typically serves veterans incarcerated in prison, and Veterans Justice Outreach (VJO), which typically serves veterans in court or in jail. There is a third VJP-aligned program, called Legal Services for Veterans, which provides grants for civil legal services (https://www.va.gov/ogc/legalservices.asp). This study was limited to veterans who received VJP outreach services from HCRV and VJO staff. Since the inception of these programs, researchers have used encounters with these programs as a broad proxy for criminal legal involvement; however, different data sources have been used to identify VJP encounters in VHA national databases. This study aimed to assess three data sources to ensure that researchers and program leaders identify the maximum number of veterans who received VJP services. By developing and validating a clear method to identify veterans in VJP, researchers can use this method to examine this patient population and develop outreach and intervention programs that may address their treatment needs. This information may also be useful for VHA clinical operations for quality monitoring and improvement to ensure this population is receiving high quality healthcare and housing services.

Although the literature documenting the clinical treatment needs among veterans with criminal legal involvement is growing [2,12], there is a need for research on veterans in VJP to improve VHA healthcare for this population. There is evidence that veterans with VJP contact received low quality care at the VHA for some conditions, including lower receipt of medications for opioid use disorder and less screening for colorectal cancer, compared to veterans without VJP contact [13–15]. The broader literature focused on people with criminal legal involvement provides limited guidance on healthcare for veterans with criminal legal involvement because of known research gaps, including exclusion of people with legal involvement from national surveys and datasets, a focus on descriptive research rather than causal modeling, and a lack of studies examining the mechanisms underlying the link between criminal legal contact and health conditions [16–18].

In the large, integrated VHA system, defining and coding a population that received specific services in VHA can be challenging because there are multiple sources of data about patients and an evolving electronic health record system [19]. Prior studies have typically used two data sources to identify VJP: (1) the VHA’s Homeless Management and Evaluation System (HOMES) database, used by the Homeless Programs Office to collect data on homeless programs including VJP [e.g., 20]; and (2) outpatient clinic stop codes of 591 for HCRV and 592 for VJO [e.g., 13]. Clinic stop codes are three-digit codes that define the clinical work units for a VHA outpatient encounter [21]. Studies using these data sources have not examined the concordance or discrepancies between sources. For example, there may be variability in how clinic stop codes are applied or missing workload documentation. Some records, such as through HOMES, may be collected more frequently in court settings when VHA staff may have more time with each veteran compared to jail settings. Different VHA medical centers may also have different conventions for using chart note titles. Electronic health record modernization may also impact documentation of VJP contact. Estimates derived from unrepresentative samples can be biased and affect the validity and reliability of the study [22]. Although we may be unable to identify veterans with criminal legal involvement in the VHA who do not have contact with VJP using the data sources we examine here, the results of this study findings will inform modernization to the VHA’s electronic health record system [23,24] and encourage the use of common data sources in research and evaluation of veterans with VJP contact.

### Current study

Before healthcare services are developed and delivered to address the specific treatment needs of individual patients, patients need to be identified for those services [25]. The primary objective of this study was to quantify overlap and under-ascertainment when using single- versus multi-source definitions to identify veterans with VJP contact. Our second objective was to provide a reproducible cohort-identification specification usable in the VHA data context for VHA researchers and VHA clinical operations. There are several benefits to evaluating and standardizing the data sources of VJP participation. For researchers, this standardized definition can be used to improve the quality and comparability of scientific studies on this population. For clinical operations, this definition can be used for quality improvement and evaluation efforts to ensure high quality healthcare.

## Materials and methods

This study was approved by the Stanford University Institutional Review Board (protocol #61776) and the Department of Veterans Affairs Palo Alto Research & Development Committee (project number FIN0008 blinded for review) under a waiver of informed consent. Data were accessed between June 4, 2024, and July 24, 2026, and the lead author and lead analyst had access to information that could identify individual participants during or after data collection.

### Data sources

Data were drawn from the VHA national electronic health record system, the Corporate Data Warehouse (CDW), which contains all administrative and clinical records of healthcare provided by the VHA. CDW includes data from the Computerized Patient Record System (CPRS), which is the longstanding VHA electronic health record system, and Millenium data, which is the healthcare record system in process of adoption across the VHA system [23,24]. Most data were from CPRS with Millenium data drawn from the VHA medical centers that have transitioned to this system, including Spokane, Walla Walla, Columbus, Roseburg, White City, and North Chicago.

Three types of files were used in this study. First, electronic health records that include clinic stop codes indicating encounters with VJP staff were compiled from workload tables. Second, the Homeless Registry, which includes the HOMES data tracking system for the VHA’s Homeless Programs Office, was used. Third, chart note titles that included terms related to the Veterans Justice Programs, Health Care for Re-entry Veterans, or Veterans Justice Outreach were used. Scrambled social security numbers were used to link the same veterans across data sources. Details about the data sources and codes from each file type are listed in Table 1 and code lists and aggregate data are included in the supporting information (S1 Table).

**Table 1:** Veterans Justice Programs Data Sources and Codes in Veterans Health Administration Electronic Health Records.

Data source type	Data source	Codes or records	Description
Clinic stop code	CDW CPRS and Millenium data	591 – HCRV 592 – VJO	Clinic stop codes for VJP outpatient encounters with veterans
HOMES record	HOMES data	HCRV entry and exit forms or VJO entry, progress, and exit forms	VJP forms collected during outreach
Clinic note title	CDW clinic note titles from charts	%Health%Care%for%Re%entry% %HCRV% %Veteran%Justice%Outreach% %VJO% %Veteran%Justice%Program% %VJP%	Clinic note titles from charts that include terms related to HCRV, VJO, or VJP

CDW = Corporate Data Warehouse. CPRS = Computerized Patient Record System. HCRV = Health Care for Re-entry Veterans. VJO = Veterans Justice Outreach. VJP = Veterans Justice Programs. HOMES = Homeless Outreach and Management Evaluation System.

### Samples

For the primary analysis examining overlap between the three data sources, all veterans who had a clinic stop code, HOMES record, or chart note title indicating contact with VJP in Fiscal Year 2024 (October 1, 2023, through September 30, 2024) were included in our sample. Veterans who were ineligible for VHA healthcare or did not have CDW electronic health records were excluded because these veterans did not have patient information necessary for the study steps examining associations between patient factors, different data sources, and diagnosis and receipt of medications for opioid use disorder (n = 929). To provide information on change in data source overlap over time, Fiscal Year 2014 (October 1, 2013, through September 30, 2014) and Fiscal Year 2019 (October 1, 2018, through September 30, 2019) were also included. Veterans who were ineligible or did not have CDW electronic health records were also excluded from these Fiscal Years (2014: n = 2,400; 2019: n = 1,342).

### Measures

#### Contact.

VJP contact was defined as the presence of any of the following in the electronic health records: (1) a VJP clinic stop code encounter of 591 or 592; (2) a HOMES HCRV or VJO entry, progress, or exit form; or (3) a chart note title with Veterans Justice Programs, Health Care for Re-entry Veterans, or Veterans Justice Outreach or related abbreviations (Table 1). HCRV contact was defined as presence of any of the following in the electronic health records: (1) a HCRV clinic stop code encounter of 591; (2) a HOMES HCRV entry or exit form; or (3) a chart note title with Health Care for Re-entry Veterans or HCRV. VJO contact was defined as presence of any of the following in the electronic health records: (1) a VJO clinic stop code of 592; (2) a HOMES VJO entry, progress, or exit form; or (3) a chart note title with Veterans Justice Outreach or VJO

### Patient and facility characteristics

Information on patient demographic and clinical characteristics was drawn from CDW electronic health records. Patient demographics included *sex* (female, male), *age* (< 35, 35–44, 45–54, and 55+), *race/ethnicity* (non-Hispanic American Indian/Alaskan Native, non-Hispanic Asian, non-Hispanic Black, non-Hispanic Multiracial, Hispanic, non-Hispanic White), *marital status* (unmarried, married) *residence* (rural, urban), and *housing status* (homeless, housed). Clinical characteristics included *service-connected disability rating* (none, < 50%, 50–100%), *any mental health disorder diagnosis* (no, yes; including anxiety, bipolar, depression, personality disorders, post-traumatic stress disorder, schizophrenia, and other mental health conditions), *any substance use disorder diagnosis* (no, yes; including alcohol, amphetamine, cannabis, cocaine, opioid, or other drug use disorders), and the Charlson comorbidity index [26]. Facility characteristics included *geographic location* (Northeast, South, Midwest, West) and *location* (rural, urban).

### Opioid use disorder diagnosis and receipt of medications

To explore the validity of the three data sources in a concrete use case, the rate of diagnosis for opioid use disorder and receipt of medications for opioid use disorder were examined. *Opioid use disorder diagnosis* was defined as any outpatient or inpatient record with an International Classification of Diseases-10 (ICD-10) code indicating opioid use disorder during Fiscal Year 2024 (S2 Table). *Receipt of medications for opioid use disorder* was defined using the American Society for Addiction Medicine’s version 2 specifications [27]. Veterans were coded as having received medications for opioid use disorder if they had at least one methadone clinic outpatient visit in the VHA or VHA-contracted care with a concurrent opioid use disorder diagnosis and/or at least one pharmacy prescription fill for oral or injectable buprenorphine or oral or injectable naltrexone during the same Fiscal Year as their opioid use disorder diagnosis (S2 Table).

### Analysis

The number of veterans with VJP contact were calculated for each data source and then the unique number of veterans with any VJP contact was calculated. For the primary analysis, the overlap between the three data sources was calculated using the Fiscal Year 2024 sample and agreement was tested using Fleiss’s kappa. To examine changes over time, data source overlaps using the Fiscal Years 2014 and 2019 samples were calculated and agreement was tested using Fleiss’s kappa. Next, using the Fiscal Year 2024 sample, the frequencies and percents for patient and facility characteristics were calculated. Three multi-level logistic regression models were used to test the association between each characteristic and receipt of a (1) clinic stop code, (2) HOMES record, or (3) chart note title. Finally, the number, percent, and 95% confidence interval of veterans diagnosed with an opioid use disorder and those who received medications for opioid use disorder were calculated. All statistical analyses were conducted in SAS version 9.04.01 [28].

## Results

### Veterans Justice Programs

In Fiscal Year 2024, there were 52,672 unique veterans who had VJP contact, as indicated by at least one data source, and who were eligible for VHA (Table 2). Overall, 80% of veterans had a clinic stop code record, 22% had a HOMES record, and 63% had a chart note title indicating contact with VJP. The most common patterns of VJP identification were clinic stop code + chart note title (41%), clinic stop code only (26%), and chart note title only (12%). A Fleiss’ kappa was calculated, κ = 0.22, *p* < .001, indicating fair evidence against the null hypothesis of no agreement [29].

**Table 2:** Patterns of Veterans Justice Programs identification by data source type and Fiscal Year.

	Fiscal Year 2014 N (%)	Fiscal Year 2019 N (%)	Fiscal Year 2024 N (%)
**Any VJP**	49,520 (100%)	54,909 (100%)	52,672 (100%)
**Clinic stop code**	**40,014 (81%)**	**48,428 (88%)**	**42,371 (80%)**
**HOMES record**	**22,686 (46%)**	**17,718 (32%)**	**11,522 (22%)**
**Chart note title**	**18,722 (38%)**	**25,969 (47%)**	**33,344 (63%)**
Clinic stop code only	14,901 (30%)	18,729 (34%)	13,541 (26%)
HOMES record only	6,247 (13%)	2,805 (5%)	3,506 (7%)
Chart note title only	2,874 (6%)	3,001 (5%)	6,198 (12%)
Clinic stop code + HOMES record	9,650 (19%)	7,406 (13%)	2,281 (4%)
Clinic stop code + chart note title	9,059 (18%)	15,461 (28%)	21,411 (41%)
HOMES record + Chart note title	385 (1%)	675 (1%)	597 (1%)
All three data sources	6,404 (13%)	6,832 (12%)	5,138 (10%)
**Any HCRV**	11,166 (100%)	7,487 (100%)	8,568 (100%)
**Clinic stop code**	**7,058 (63%)**	**6,254 (84%)**	**7,270 (85%)**
**HOMES record**	**7,754 (69%)**	**3,154 (42%)**	**1,685 (20%)**
**Chart note title**	**960 (9%)**	**1,200 (16%)**	**2,053 (24%)**
Clinic stop code only	2,919 (26%)	3,585 (48%)	5,183 (60%)
HOMES record only	3,800 (34%)	997 (13%)	625 (7%)
Chart note title only	238 (2%)	205 (3%)	631 (7%)
Clinic stop code + HOMES record	3,487 (31%)	1,705 (23%)	707 (8%)
Clinic stop code + chart note title	255 (2%)	543 (7%)	1,069 (12%)
HOMES record + chart note title	70 (1%)	31 (<1%)	42 (1%)
All three data sources	397 (4%)	421 (6%)	311 (4%)
**Any VJO**	39,494 (100%)	48,731 (100%)	46,294 (100%)
**Clinic stop code**	**33,551 (85%)**	**43,097 (88%)**	**36,595 (79%)**
**HOMES record**	**15,138 (38%)**	**14,659 (30%)**	**9,970 (22%)**
**Chart note title**	**17,452 (44%)**	**24,490 (50%)**	**28,660 (62%)**
Clinic stop code only	12,920 (33%)	16,364 (34%)	12,223 (26%)
HOMES record only	2,854 (7%)	2,036 (4%)	3,222 (7%)
Chart note title only	2,777 (7%)	2,948 (6%)	6,015 (13%)
Clinic stop code + HOMES record	6,268 (16%)	5,841 (12%)	2,189 (5%)
Clinic stop code + chart note title	8,659 (22%)	14,760 (30%)	18,086 (39%)
HOMES record + chart note title	312 (1%)	650 (1%)	462 (1%)
All three data sources	5,704 (14%)	6,132 (13%)	4,097 (9%)

Note: **Bold** rows indicate data sources with overlap. VJP = Veterans Justice Programs. HOMES = Homeless Operations and Management Evaluation System. HCRV = Health Care for Re-entry Veterans. VJO = Veterans Justice Outreach.

**Fig 1:**
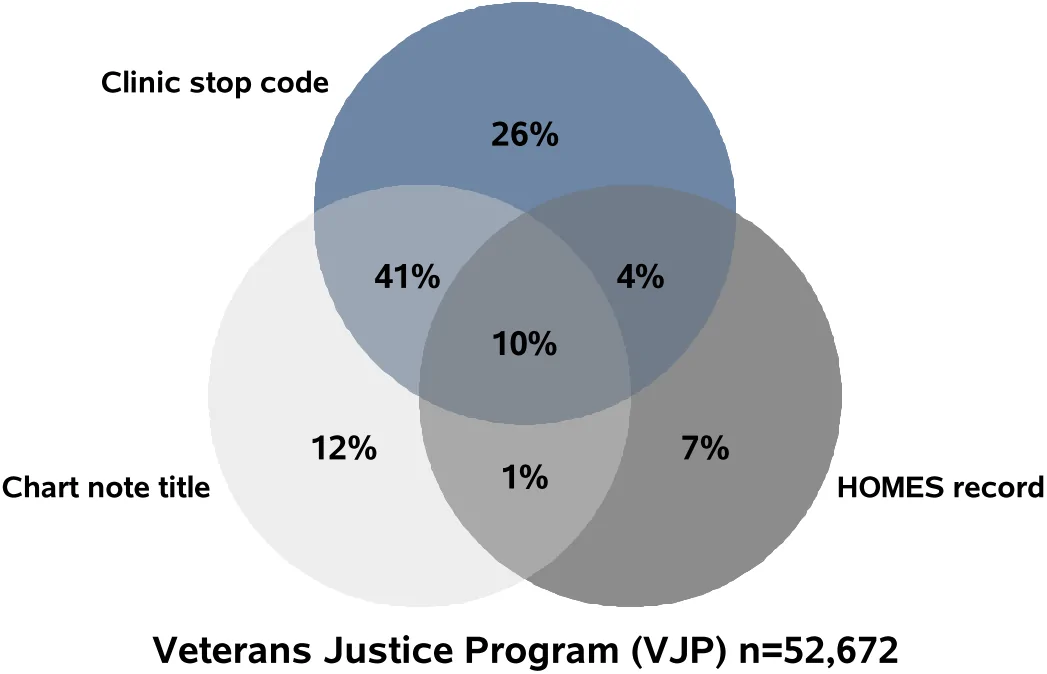
Overlap by data source in veterans with Veterans Justice Programs contact in Fiscal Year 2024.

The overlap in data source types for VJP is depicted in Fig 1 using Fiscal Year 2024 data. The blue shaded circle (top of Fig 1) indicates veterans who received VJP clinic stop codes. The dark gray circle (bottom right of Fig 1) indicates veterans who received a VJP HOMES record. The light gray circle (bottom left of Fig 1) indicates who received a VJP chart note title. The clinic stop code circle indicates that 26% of veterans received a VJP clinic stop code only, 4% received both a clinic stop code and a HOMES record, and 41% received both a clinic stop code and a chart note title. Only 10% of veterans had records of all three data sources (center overlap of all circles).

The Fiscal Years 2014 and 2019 samples indicated that the percentage of data sources changed over time (Table 2). There was some variation in the percentage of veterans who received a clinic stop code in any VJP, but at least 80% did during any of the three Fiscal Years examined. The percentage of veterans who received a HOMES record declined over time from 46% in Fiscal Year 2014, to 32% in Fiscal Year 2019, and 22% in Fiscal Year 2024. Conversely, the percentage of veterans who received a chart note title increased from 38% in Fiscal Year 2014 to 47% in Fiscal Year 2019 and 63% in Fiscal Year 2024. The same overall pattern was reflected in the subgroups with receipt of a HOMES record declining over time and receipt of a chart note title increasing over time.

### Health Care for Reentry Veterans

In Fiscal Year 2024, there were 8,568 veterans who had contact with the HCRV program and were eligible for VHA services (Table 2). Of these veterans, there were 2,522 (29%) who also had contact with the VJO program. Overall, most veterans (85%) had a clinic stop code record, whereas less than a quarter had a HOMES record (20%) or a chart note title (24%) indicating contact with HCRV. The most common patterns of HCRV identification were clinic stop code only (60%), and clinic stop code + chart note title (12%). A Fleiss’ kappa was calculated, κ = 0.27, *p* < .001, indicating fair evidence against the null hypothesis of no agreement.

**Fig 2:**
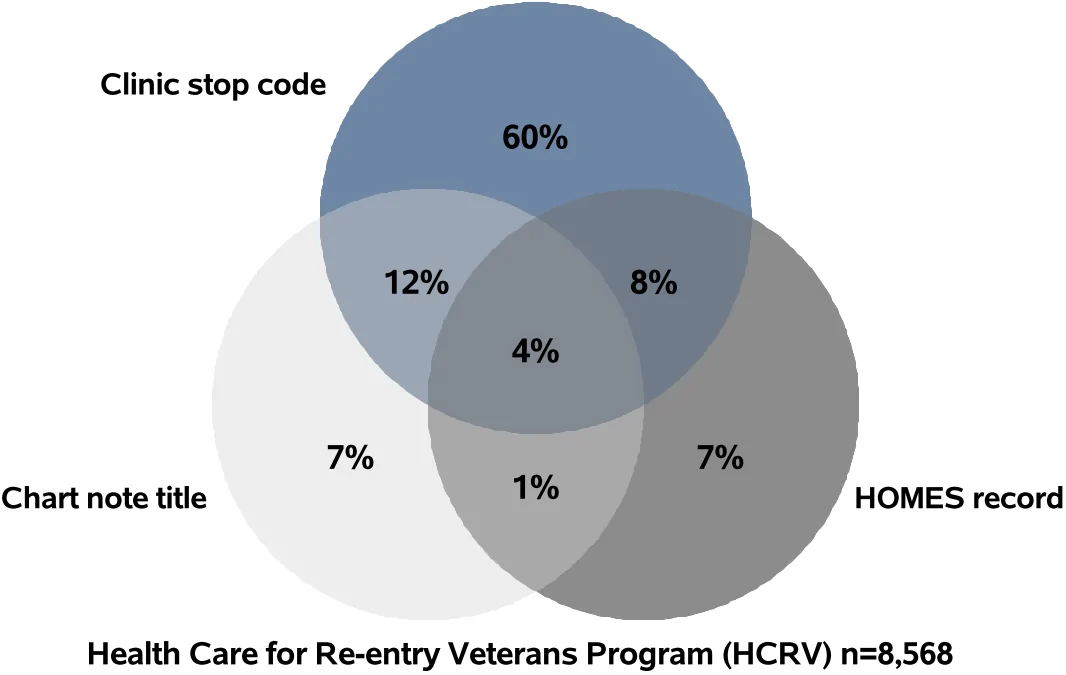
Overlap by data source in veterans with Health Care for Re-entry Veterans contact in Fiscal Year 2024.

The overlap in data source types for HCRV is depicted in Fig 2 using Fiscal Year 2024 data. The blue shaded circle (top of Fig 2) indicates veterans who received a HCRV clinic stop code. The dark gray circle (bottom right of Fig 2) indicates veterans who received a HCRV HOMES record. The light gray circle (bottom left of Fig 2) indicates who received a HCRV chart note title. The HOMES record circle indicates that 7% of veterans received a HCRV HOMES record only, 8% received both a HOMES record and a HCRV clinic stop code, and 1% received both a HCRV HOMES record and a HCRV chart note title. Only 4% had all three types of data sources (center overlap of all circles).

For HCRV, receipt of a clinic stop code or a chart note title increased over time (Table 2). For example, 63% of veterans received a clinic stop code in Fiscal Year 2014, 84% in Fiscal Year 2019, and 85% in Fiscal Year 2024. Chart note title receipt increased from 9% in Fiscal Year 2014 to 16% in Fiscal Year 2019 and to 24% in Fiscal Year 2024. Receipt of a HOMES record declined over time from 69% in Fiscal Year 2014 to 42% in Fiscal Year 2019 and 20% in Fiscal Year 2024.

### Veterans Justice Outreach

In Fiscal Year 2024, there were 46,294 veterans who had contact with the VJO program and were eligible for VHA (Table 2). Most veterans (79%) had a VJO clinic stop code record, whereas less than half had a VJO HOMES record (22%). Over half (62%) had a VJO chart note title. The most common VJO identification patterns were clinic stop code + chart note title (39%), clinic stop code only (26%), and chart note title only (13%). A Fleiss’ kappa was calculated, κ = 0.22, *p* < .001, indicating fair evidence against the null hypothesis of no agreement.

**Fig 3:**
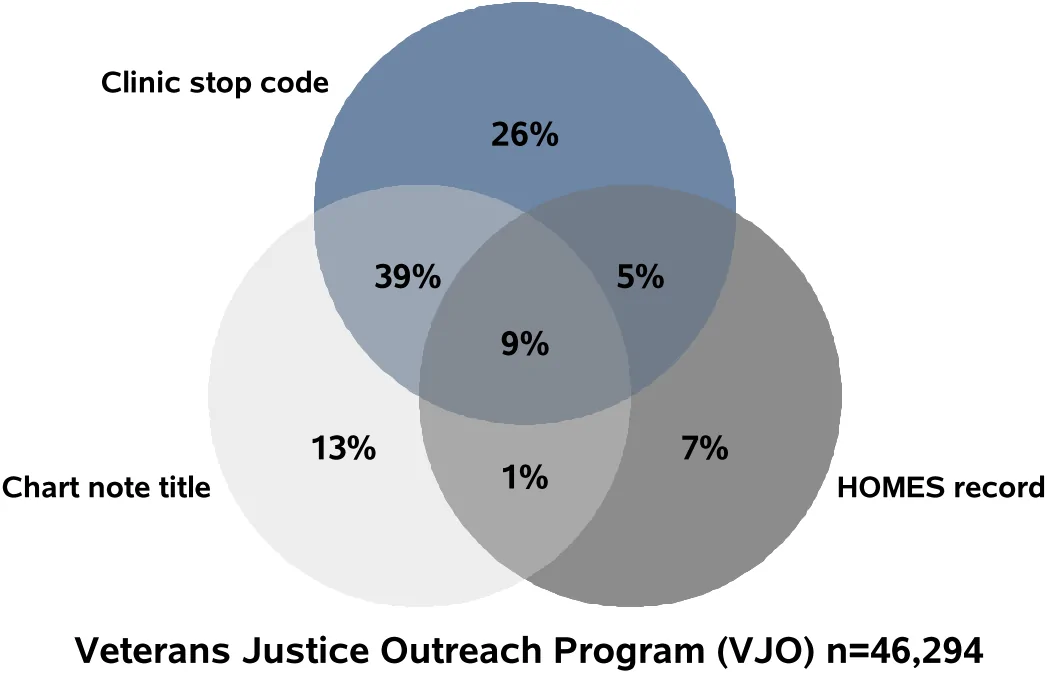
Overlap by data source in veterans with Veterans Justice Outreach contact in Fiscal Year 2024.

The overlap in data source types for VJO is depicted in Fig 3 using Fiscal Year 2024 data. The blue shaded circle (top of Fig 3) indicates veterans who received a VJO clinic stop code. The dark gray circle (bottom right of Fig 3) indicates veterans who received a VJO HOMES record. The light gray circle (bottom left of Fig 3) indicates who received a VJO chart note title. The chart note circle indicates that 13% of veterans received a VJO chart note title only, 39% received both a VJO chart note title and a VJO clinic stop code, and 1% received both a VJO chart note title and a VJO HOMES record. Only 9% had all three types of data sources (center overlap of all circles).

The historical pattern of VJO data sources is reported in Table 2. In Fiscal Year 2014, 85% of veterans received a clinic stop code compared to 88% in Fiscal Year 2019 and 79% in Fiscal Year 2024. Receipt of a HOMES record declined from 38% in Fiscal Year 2014 to 30% in Fiscal Year 2019 and 22% in Fiscal Year 2024. Receipt of a chart note increased from 44% in Fiscal Year 2014 to 50% in Fiscal Year 2019 and 62% in Fiscal Year 2024.

### Patient and facility factors associated with identification data source

Patient factors varied by the type of data source, as indicated by the logistic regression models reported in Table 3. For example, female veterans comprised 7% of the sample and had lower odds of having a HOMES record (adjusted odds ratio [AOR] = 0.75, 95% confidence interval [CI] = 0.63–0.91, *p* = .003) but higher odds of having a chart note title (AOR = 1.32, 95% CI = 1.17–1.49, *p* < .001) compared to male veterans. Compared to veterans under age 35, veterans age 35 and older had lower odds of receiving a clinic stop code and higher odds of receiving a HOMES record. Similarly, unmarried veterans had lower odds of receiving a clinic stop code (AOR = 0.88, 95% CI = 0.83–0.94, *p* < .001) and higher odds of receiving a HOMES record (AOR = 1.34, 95% CI = 1.23–1.47, *p* < .001) compared to veterans who were married. Veterans who lived in rural areas compared to urban (AOR = 0.86, 95% CI 0.75–0.98, *p* = .028) and veterans who were homeless compared to housed (AOR = 0.68, 95% CI = 0.56–0.83, *p* < .001) had lower odds of receiving a HOMES record.

**Table 3:** Patient and facility factors associated with Veterans Justice Programs identification by data source in Fiscal Year 2024.

		Clinic stop code		HOMES record		Chart note title	
**Patient characteristics**	N (%)	AOR (95% CI)	*p*	AOR (95% CI)	*p*	AOR (95% CI)	*p*
Sex							
Female	3,862 (7%)	0.91 (0.82, 1.02)	.122	0.75 (0.63, 0.91)	.003	1.32 (1.17, 1.49)	< .001
Male	45,876 (87%)	ref		ref		ref	
Missing	2,934 (6%)	–		–		–	
Age							
< 35	10,098 (19%)	ref		ref		ref	
35-44	14,450 (27%)	0.80 (0.72, 0.90)	< .001	1.64 (1.36, 1.98)	< .001	1.01 (0.87, 1.19)	.817
45-54	8,685 (16%)	0.78 (0.69, 0.87)	< .001	1.66 (1.38, 1.99)	< .001	1.06 (0.91, 1.24)	.407
55+	19,439 (37%)	0.68 (0.60, 0.77)	< .001	1.68 (1.41, 2.00)	< .001	1.29 (1.10, 1.52)	.002
Race/ethnicity							
American Indian/ Alaskan Native	742 (1%)	1.25 (0.98, 1.58)	.061	0.73 (0.52, 1.02)	.070	0.86 (0.64, 1.16)	.329
Asian	832 (2%)	1.19 (0.95, 1.50)	.127	0.84 (0.59, 1.19)	.341	0.85 (0.62, 1.16)	.320
Black	12,485 (24%)	1.02 (0.93, 1.13)	.603	0.97 (0.84, 1.12)	.685	0.98 (0.87, 1.09)	.727
Hispanic	5,256 (10%)	1.02 (0.90, 1.15)	.754	1.05 (0.88, 1.25)	.574	0.93 (0.79, 1.09)	.390
Multiracial	579 (1%)	0.97 (0.72, 1.31)	.888	1.17 (0.80, 1.73)	.408	0.92 (0.60, 1.42)	.731
White	29,834 (57%)	ref		ref		ref	
Missing	2,944 (6%)	–		–		–	
Marital status							
Unmarried	34,885 (66%)	0.88 (0.83, 0.94)	< .001	1.34 (1.23, 1.47)	< .001	0.99 (0.91, 1.08)	.920
Married	12,992 (25%)	ref		ref		ref	
Missing	4,795 (9%)	–		–		–	
Residence							
Rural	12,095 (23%)	1.06 (0.95, 1.18)	.293	0.86 (0.75, 0.98)	.028	1.00 (0.85, 1.18)	.975
Urban	39,772 (76%)	ref		ref		ref	
Missing	805 (2%)	–		–		–	
Housing status							
Homeless	17,081 (32%)	1.09 (0.96, 1.24)	.173	0.68 (0.56, 0.83)	< .001	1.09 (0.94, 1.26)	.227
Housed	35,591 (68%)	ref		ref		ref	
Service-connected disability rating							
None	17,903 (34%)	ref		ref		ref	
< 50%	7,293 (14%)	0.82 (0.75, 0.89)	< .001	1.36 (1.19, 1.54)	< .001	1.11 (0.99, 1.25)	.054
50-100%	27,476 (52%)	0.80 (0.74, 0.86)	< .001	1.46 (1.26, 1.69)	< .001	1.10 (1.00, 1.22)	.037
Any mental health diagnosis							
Yes	34,188 (65%)	0.84 (0.77, 0.93)	.001	1.00 (0.87, 1.15)	.979	1.29 (1.16, 1.44)	< .001
No	18,484 (35%)	ref		ref		ref	
Any substance use disorder diagnosis							
Yes	24,854 (47%)	1.21 (1.10, 1.33)	< .001	0.76 (0.67, 0.88)	< .001	0.87 (0.78, 0.97)	.015
No	27,818 (53%)	ref		ref		ref	
Comorbidity index, mean (SD)	0.68 (1.57)	0.95 (0.93, 0.96)	< .001	1.03 (1.01, 1.06)	.002	1.05 (1.03, 1.07)	< .001
**Facility characteristics**							
Geographic location							
Northeast	6,782 (13%)	ref		ref		ref	
South	19,690 (37%)	1.00 (0.59, 1.69)	.977	0.71 (0.35, 1.43)	.344	1.73 (0.61, 4.84)	.296
Midwest	11,282 (21%)	0.74 (0.41, 1.31)	.306	0.92 (0.43, 1.96)	.829	1.81 (0.53, 6.19)	.342
West	14,918 (28%)	0.66 (0.30, 1.45)	.305	0.55 (0.25, 1.23)	.150	3.51 (0.96, 12.80)	.056
Location							
Rural	12,373 (24%)	0.93 (0.59, 1.47)	.771	0.96 (0.59, 1.54)	.866	1.39 (0.59, 3.27)	.447
Urban	40,299 (77%)	ref		ref		ref	

*Note*. Missing values were not included in the models. HOMES = Homeless Operations and Management Evaluation System. AOR = adjusted odds ratio. CI = confidence interval. Ref = reference group. SD = standard deviation.

Patient clinical characteristics varied in their association with type of data source. Compared to veterans with no service-connected disability rating, veterans with any service-connected disability rating had lower odds of receiving a clinic stop code and higher odds of receiving a HOMES record (Table 3). Veterans with any mental health diagnosis had lower odds of receiving a clinic stop code (AOR = 0.84, 95% CI = 0.77–0.93, *p* = .001) and higher odds of receiving a chart note title (AOR = 1.29, 95% CI = 1.16–1.44, *p* < .001) compared to veterans with no mental health diagnosis. The opposite pattern was found for veterans with any substance use disorder diagnosis who had higher odds of receiving a clinic stop code (AOR = 1.21, 95% CI = 1.10–1.33, *p* < .001) and lower odds of receiving a chart note (AOR = 0.87, 95% CI = 0.78–0.97, *p* = .015) than veterans with no substance use disorder diagnosis. For Fiscal Year 2014, there was no significant differences by service-connected disability rating or mental health diagnosis, but a similar pattern was observed for substance use disorder diagnosis (i.e., veterans with a substance use disorder diagnosis had higher odds or receiving a clinic stop code and lower odds of receiving a chart note title; S3 Table). For Fiscal Year 2019, the pattern of results was similar to Fiscal Year 2024 (S4 Table).

### Medications for Opioid Use Disorder

The rate of opioid use disorder diagnosis and receipt of medications for opioid use disorder differed depending on the data source used to identify veterans in VJP (Table 4). When clinic stop codes were used to identify veterans with VJP contact, 7.5% of the sample (95% CI = 7.3%−7.8%) had an opioid use disorder diagnosis, whereas 10.9% (95% CI = 10.3–11.5%) had an opioid use disorder diagnosis when HOMES records were used to identify VJP contact. The more granular non-overlapping groups also showed differences. For example, the clinic stop codes only group had an opioid use disorder diagnosis rate of 5.3% (95% CI = 4.9–5.7%) whereas the all three data sources group had an opioid use disorder diagnosis rate of 13.8% (95% CI = 12.9–14.8%). Similarly, for receipt of medications for opioid use disorder, the HOMES records only group had a receipt rate of 51.6% (95% CI = 44.8–58.3%) compared to the HOMES records + chart note titles group with a receipt rate of 57.1% (95% CI = 43.2–70.3%).

**Table 4:** Rate of opioid use disorder diagnosis and receipt of medications for opioid use disorder in Fiscal Year 2024 by Veterans Justice Program identification group.

Group	Sample size	Opioid use disorder diagnosis		Receipt of medications for opioid use disorder	
	N (%)	N (%)	95% CI	N (%)	95% CI
Any data source	52,672 (100%)	3,901 (7.4%)	(7.2%, 7.6%)	2,115 (54.2%)	(52.6%, 55.8%)
Clinic stop codes	42,371 (81%)	3,180 (7.5%)	(7.3%, 7.8%)	1,739 (54.7%)	(52.9%, 56.4%)
HOMES records	11,522 (22%)	1,252 (10.9%)	(10.3%, 11.5%)	685 (54.7%)	(51.9%, 57.5%)
Chart note titles	33,344 (64%)	2,692 (8.1%)	(7.8%, 8.4%)	1,491 (55.4%)	(53.5%, 57.3%)
Clinic stop codes only	13,541 (26%)	723 (5.3%)	(4.9%, 5.7%)	374 (51.7%)	(48.0%, 55.4%)
HOMES records only	3,506 (7%)	223 (6.4%)	(5.6%, 7.2%)	115 (51.6%)	(44.8%, 58.3%)
Chart note titles only	6,198 (12%)	442 (7.1%)	(6.5%, 7.8%)	229 (51.8%)	(47.0%, 56.6%)
Clinic stop codes + HOMES records	2,281 (4%)	263 (11.5%)	(10.3%, 12.9%)	135 (51.3%)	(45.1%, 57.5%)
Clinic stop codes + Chart note titles	21,411 (41%)	1,484 (6.9%)	(6.6%, 7.3%)	827 (55.7%)	(53.2%, 58.3%)
HOMES records + Chart note titles	597 (1%)	56 (9.4%)	(7.2%, 12.0%)	32 (57.1%)	(43.2%, 70.3%)
All three data sources	5,138 (10%)	710 (13.8%)	(12.9%, 14.8%)	403 (56.8%)	(53.0%, 60.4%)

CI = confidence interval. HOMES = Homeless Outreach Management and Evaluation System.

## Discussion

This methodological study provides evidence that identification of veterans who received VJP services differs depending on the data source used. Furthermore, the proportion of veterans identified by each data source shifts over time, with use of HOMES records declining from Fiscal Years 2014 to 2024 and use of chart note titles increasing during that same period. Finally, depending on the data source used, the patients identified, the prevalence of a clinical diagnosis, and the receipt of high-quality healthcare will all differ. The results suggest the use of all three data sources, when possible and appropriate, is the most comprehensive strategy of those examined in this study. We include information about code lists in the Supporting Information.

Although there may be reasons why researchers choose to use certain data sources, use of the three data sources is encouraged when the goal is to identify the maximum number of veterans with VJP contact. For example, some studies use HOMES records to identify veterans in VJP [20]. The HOMES data may be preferrable to answer some research questions because more detailed information not routinely captured in other VHA patient records is collected, including type of crime, recent living situation, employment and income, and program discharge information. Using HOMES records in Fiscal Year 2014 identified 46% of the veterans who had HCRV contact. However, women and Hispanic veterans were less represented in HOMES records at that time. Only 22% of veterans received a HOMES record in Fiscal Year 2024, which introduces difficulties with observing changes over time. Until VHA develops universal data documentation standards for these veterans, use of the three suggested data sources may help provide comparability over time and across studies, rather than use of one or two of the data sources. Likewise, the clinic stop codes are the simplest to define and will identify about 80% of veterans who received VJP services, but there may be systematic bias or logistical administrative reasons in who did or did not receive these codes, which has implications for clinical care. Examining Fiscal Year 2024 data, if clinic stop codes were used, 7.5% of veterans were diagnosed with opioid use disorder compared to 10.9% when HOMES records are used and 8.1% when chart note titles are used. The results suggest that even when using the largest data source (clinic stop codes), there is still value added from incorporating additional data sources (HOMES and chart note titles) to identify veterans with VJP contact.

For clinical operations, these three data sources can be used to examine veterans with VJP contact over time to ensure previously identified healthcare quality gaps are remedied. Correct identification of the patient population is necessary for this quality improvement work [30]. For example, colorectal cancer screening is lower among veterans with VJP contact compared to other veterans served at VHA [15]. Efforts are underway to improve colorectal cancer screening rates for the entire population of veterans who meet criteria. If veterans with VJP contact continue to have worse quality of care, more targeted quality improvement programming can be developed. Clinical operations leaders may also improve existing VHA interventions programs by incorporating these data sources to identify veterans with VJP contact. For example, the VHA predictive model that identifies veterans at high risk for suicide death could be improved by including data on veterans with VJP contact [31].

The results of this study are generalizable in two ways. First, these methods may serve as a model for researchers who study other populations in VHA that can be identified from multiple data sources, such as clinic stop codes and chart note titles. Second, although the specifics of the codes we examined are not generalizable to other healthcare systems because they do not have the same data sources, the overall method may be helpful to healthcare systems exploring ways to identify patients with criminal legal involvement using other indicators and data sources. However, we will caution that identification of patients with criminal legal involvement may have unintended negative consequences [25]. People with criminal legal involvement noted in their healthcare records may experience real or perceived discrimination by healthcare workers [32,33]. VJP guidance states that only information relevant to treatment should be included in a veteran’s medical record and details that would create stigma and barriers to treatment should be avoided [34], indicating a clear understanding of the potential negative issues around identifying veterans with criminal legal involvement. Any research or clinical operations work that utilizes these data sources to identify veterans with VJP contact should take care to minimize any real or perceived discrimination veterans may experience from healthcare workers [32,33]. Other principles that can help limit stigma in the medical record include role-based access and purpose limitation, such as limiting this information to VJP staff only or on a time-limited basis to other clinical staff.

### Limitations

There were a few limitations of our study. We do not have a gold standard data source of criminal legal involvement to compare with our findings and, therefore, we are cautious not to be prescriptive. There may have been some veterans who were mistakenly coded as having received VJP services (likely a small group) and there may be veterans with criminal legal involvement that did not use VJP services or are not in the VHA (likely a larger group) that were not identified by any of the three sources. This study was limited to evaluating the concordance among existing data sources rather than diagnostic accuracy. Future validation against external criminal legal system data, where feasible, would further strengthen confidence in the proposed identification strategy; however, such analyses would require data from law enforcement, courts, jails, prisons, and community supervision departments. Research exploring alternative definitions, such as those using ICD-10 codes indicating legal involvement, will also be valuable. Analyses were restricted to veterans who were eligible for VHA services and had a CDW record with 929 veterans with VJP contact in Fiscal Year 2024 excluded. Depending on the intended use of the identification method, other studies may want to include veterans who are ineligible for VHA care or lack electronic health records. Although we included Millennium sites in our analyses, we did not examine differences by systems; therefore, bias may be present in these data depending on how clinic stop codes and chart note titles are utilized in Millennium sites. Finally, our methodology for identifying veterans will not generalize to other healthcare systems because they do not have VJP services.

## Conclusions

Single-source ascertainment under-identified VJP contact. A standardized multi-source definition increases data capture of veterans with any VJP contact compared to other approaches evaluated in this study. Veterans with different demographic and clinical characteristics are identified differently depending on the data source used. The multi-source definition provided in this study was more comprehensive than single-source definitions and may be useful to VHA stakeholders focused on identifying the maximum number of veterans with VJP contact.

## Supporting information

S1 Table Data sources and tables. (DOCX)

S2 Table Specifications for opioid use disorder diagnosis and medications. (DOCX)

S3 Table Patient and facility characteristics associated with Veterans Justice Programs identification by data source in Fiscal Year 2014. (DOCX)

S4 Table Patient and facility characteristics associated with Veterans Justice Programs identification by data source in Fiscal Year 2019. (DOCX)

## References

[1] Maruschak LM, Bronson J, Alper M. Survey of prison inmates. Veterans in prison. Washington, DC: U.S. Department of Justice, Office of Justice Programs, Bureau of Justice Statistics. 2021.

[2] Singh K, Timko C, Yu M, Taylor E, Blue-Howells J, Finlay AK. Scoping review of military veterans involved in the criminal legal system and their health and healthcare: 5-year update and map to the Veterans-Sequential Intercept Model. Health Justice. 2024;12(1):18. doi: 10.1186/s40352-024-00274-9 38639813 PMC11027330

[3] Finlay AK, Palframan KM, Stimmel M, McCarthy JF. Legal system involvement and opioid-related overdose mortality in u.s. department of veterans affairs patients. Am J Prev Med. 2022;62(1):e29–37. doi: 10.1016/j.amepre.2021.06.014 34521559 PMC8849578

[4] Kelton K, Yu M, Singh K, Harris AHS, Blue-Howells J, Stimmel M, et al. Risk of homelessness among veterans with and without criminal legal system involvement. Psychol Serv. 2025;:10.1037/ser0000993. doi: 10.1037/ser0000993 40875388 PMC12396513

[5] Palframan KM, Blue-Howells J, Clark SC, McCarthy JF. Veterans justice programs: assessing population risks for suicide deaths and attempts. Suicide Life Threat Behav. 2020;50(4):792–804. doi: 10.1111/sltb.12631 32147866

[6] Edwards ER, Fortuna A, Holliday R, Addison H, Tsai J. Prevalence of mental health conditions, substance use disorders, suicidal ideation and attempts, and experiences of homelessness among Veterans with criminal-legal involvement: A meta-analysis. Clin Psychol Rev. 2025;115:102533. doi: 10.1016/j.cpr.2024.102533 39740354

[7] Elbogen EB, DuBois CM, Finlay AK, Clark S, Kois LE, Wagner HR, et al. How often does homelessness precede criminal arrest in veterans? Results from the U.S. survey of prison inmates. Am J Orthopsychiatry. 2023;93(6):486–93. doi: 10.1037/ort0000693 37561476

[8] Tsai J, Rosenheck RA. Psychosis, Lack of Job Skills, and Criminal History: Associations With Employment in Two Samples of Homeless Men. Psychiatr Serv. 2016;67(6):671–5. doi: 10.1176/appi.ps.201500145 26766756

[9] Han BH, Bronson J, Washington L, Yu M, Kelton K, Tsai J, et al. Co-occurring Medical Multimorbidity, Mental Illness, and Substance Use Disorders Among Older Criminal Legal System-Involved Veterans. Med Care. 2023;61(7):477–83. doi: 10.1097/MLR.0000000000001864 37204150 PMC10330246

[10] LePage JP, Bradshaw LD, Cipher DJ, Crawford AM, Parish-Johnson JA. The association between recent incarceration and inpatient resource use and death rates: evaluation of a US veteran sample. Public Health. 2016;134:109–13. doi: 10.1016/j.puhe.2016.01.009 26903396

[11] Blue-Howells JH, Clark SC, van den Berk-Clark C, McGuire JF. The U.S. Department of Veterans Affairs Veterans Justice programs and the sequential intercept model: case examples in national dissemination of intervention for justice-involved veterans. Psychol Serv. 2013;10(1):48–53. doi: 10.1037/a0029652 22924802

[12] Finlay AK, Owens MD, Taylor E, Nash A, Capdarest-Arest N, Rosenthal J, et al. A scoping review of military veterans involved in the criminal justice system and their health and healthcare. Health Justice. 2019;7(1):6. doi: 10.1186/s40352-019-0086-9 30963311 PMC6718001

[13] Finlay AK, Harris AHS, Rosenthal J, Blue-Howells J, Clark S, McGuire J, et al. Receipt of pharmacotherapy for opioid use disorder by justice-involved U.S. Veterans Health Administration patients. Drug Alcohol Depend. 2016;160:222–6. doi: 10.1016/j.drugalcdep.2016.01.013 26832998 PMC4767599

[14] Finlay AK, Harris AHS, Timko C, Yu M, Smelson D, Stimmel M, et al. Disparities in Access to Medications for Opioid Use Disorder in the Veterans Health Administration. J Addict Med. 2021;15(2):143–9. doi: 10.1097/ADM.0000000000000719 32826617 PMC7889740

[15] Nieser KJ, Harris AHS, Binswanger IA, Clark SC, Finlay AK. Legal-involved veterans are less likely to receive guideline-concordant colorectal cancer screening. BMC Health Serv Res. 2025;25(1):333. doi: 10.1186/s12913-025-12490-6 40038662 PMC11877804

[16] Cao M, Esposito M, Lee H. The U.S. criminal legal system and population health. Curr Epidemiol Rep. 2025;12:5. doi: 10.1007/s40471-025-00358-6 40689265 PMC12276886

[17] Ahalt C, Binswanger IA, Steinman M, Tulsky J, Williams BA. Confined to ignorance: the absence of prisoner information from nationally representative health data sets. J Gen Intern Med. 2012;27(2):160–6. doi: 10.1007/s11606-011-1858-7 21922160 PMC3270223

[18] Wang EA, Macmadu A, Rich JD. Examining the Impact of Criminal Justice Involvement on Health Through Federally Funded, National Population-Based Surveys in the United States. Public Health Rep. 2019;134(1_suppl):22S-33S. doi: 10.1177/0033354918824324 31059413 PMC6505319

[19] Tsai J, Szymkowiak D, Jutkowitz E. Developing an operational definition of housing instability and homelessness in Veterans Health Administration’s medical records. PLoS One. 2022;17(12):e0279973. doi: 10.1371/journal.pone.0279973 36584201 PMC9803152

[20] Tsai J, Flatley B, Kasprow WJ, Clark S, Finlay A. Diversion of Veterans With Criminal Justice Involvement to Treatment Courts: Participant Characteristics and Outcomes. Psychiatr Serv. 2017;68(4):375–83. doi: 10.1176/appi.ps.201600233 27903139 PMC5752436

[21] Department of Veterans Affairs. Fiscal Year 2000 Decision Support System (DSS) Outpatient Identifiers. Washington DC: Department of Veterans Affairs. 2000.

[22] Agiwal V, Chaudhuri S. Methods and implications of addressing missing data in health-care research. Current Medical Issues. 2024;22(1):60–2. doi: 10.4103/cmi.cmi_121_23

[23] Cogan AM, Rinne ST, Weiner M, Simon S, Davila J, Yano EM. Using Research to Transform Electronic Health Record Modernization: Advancing a VA Partnered Research Agenda to Increase Research Impacts. J Gen Intern Med. 2023;38(Suppl 4):965–73. doi: 10.1007/s11606-023-08289-y 37798575 PMC10593706

[24] White PJ, Nebeker JR. Evidence Enlightens Electronic Health Record Modernization. J Gen Intern Med. 2023;38(Suppl 4):937–9. doi: 10.1007/s11606-023-08332-y 37798589 PMC10593715

[25] Armstrong N, Sutton E, Chew S, Tarrant C. Identifying patients with additional needs isn’t enough to improve care: harnessing the benefits and avoiding the pitfalls of classification. BMJ Qual Saf. 2024;33(3):152–5. doi: 10.1136/bmjqs-2023-016809 38135496

[26] Quan H, Sundararajan V, Halfon P, Fong A, Burnand B, Luthi J-C, et al. Coding algorithms for defining comorbidities in ICD-9-CM and ICD-10 administrative data. Med Care. 2005;43(11):1130–9. doi: 10.1097/01.mlr.0000182534.19832.83 16224307

[27] Harris AHS, Weisner CM, Chalk M, Capoccia V, Chen C, Thomas CP. Specifying and Pilot Testing Quality Measures for the American Society of Addiction Medicine’s Standards of Care. J Addict Med. 2016;10(3):148–55. doi: 10.1097/ADM.0000000000000203 26933875 PMC5001552

[28] SAS Institute Inc. SAS/ACCESS® 9.4 Interface to ADABAS: Reference. Cary, NC: SAS Institute Inc. 2013.

[29] Landis JR, Koch GG. The measurement of observer agreement for categorical data. Biometrics. 1977;33(1):159–74. doi: 10.2307/2529310 843571

[30] Huang T, Socrates V, Gilson A, Safranek C, Chi L, Wang EA, et al. Identifying incarceration status in the electronic health record using large language models in emergency department settings. J Clin Transl Sci. 2024;8(1):e53. doi: 10.1017/cts.2024.496 38544748 PMC10966832

[31] Meerwijk EL, Finlay AK, Harris AHS. Retraining the veterans health administration’s REACH VET suicide risk prediction model for patients involved in the legal system. Npj Ment Health Res. 2025;4(1):29. doi: 10.1038/s44184-025-00143-9 40640451 PMC12246187

[32] Frank JW, Wang EA, Nunez-Smith M, Lee H, Comfort M. Discrimination based on criminal record and healthcare utilization among men recently released from prison: a descriptive study. Health Justice. 2014;2:6. doi: 10.1186/2194-7899-2-6 25642407 PMC4308970

[33] Redmond N, Aminawung JA, Morse DS, Zaller N, Shavit S, Wang EA. Perceived Discrimination Based on Criminal Record in Healthcare Settings and Self-Reported Health Status among Formerly Incarcerated Individuals. J Urban Health. 2020;97(1):105–11. doi: 10.1007/s11524-019-00382-0 31628588 PMC7010870

[34] Veterans Justice Programs. Washington DC: Veterans Health Administration. 2024.

