# Supplementary material for: Assessing methods to identify Veterans Health Administration populations with Veterans Justice Programs contact to improve health research
Source: PLoS One. 2026 Sep 8;21(9):e0357665. doi: 10.1371/journal.pone.0357665 (PMC13552712; doi:10.1371/journal.pone.0357665)
Supplement: Supplementary file 1 [file pone.0357665.s001.docx]

**S1 Table. Data sources and tables**

| **Data source** | **Stopcode CPRS** | **Stopcode Millennium** | **HOMES** | **Chart note – CPRS** | **Chart note – Millennium** |
| --- | --- | --- | --- | --- | --- |
| CDW tables | outpat.visit dim.stopcode | billingmill_chargeitem, billingmill_billedcharge, billingmill_generalledger | HCRV entry and exit tables, VJO entry, progress, and exit tables | tiu_tiudocument, dim_TIUDocument Definition, dim_tiustandardtitle | clinicaleventmill_clinicalevent, pathologymill_discretetaskassay |
| CDW fields | visit.visitdatetime, dim.stopcode | billingmill_chargeitem.activeindicator, billingmill_chargeitem.offsetchargeitemsid, billingmill_chargeitem.servicedatetime, billingmill_billedcharge.activeindicator, billingmill_generalledger.reclassifiedindicator, billingmill_generalledger.accounttypecd | txtDtFormCompleted | dim_tiudocumentdefition.TIUDocumentDefinition, dim_tiudocumentdefinition.TIUDocumentDefinitionprintname, dim_tiudocumentdefintion.TIUDocumentDefinitionabbreviation, dim_tiustandardtitle.TIUStandardTitle, tiu_tiudocument.entrydatetime | clinicaleventmill_clinicalevent.event, pathologymill_discretetaskassay.event, clincialeventmill_clinicalevent.event.eventenddatetime |
| Inclusion filters | primarystopcode in (591,592) or secondarystopcode in (591,592) | billingmill_chargeitem.activeindicator=1, billingmill_chargeitem.offsetchargeitemsid<1, billingmill_chargeitem.servicedatetime between ‘2023-10-01’ and ‘2024-09-30, billingmill_billedcharge.activeindicator=1, billingmill_generalledger.reclassifiedindicator=0, billingmill_generalledger.accounttypecd=’649382’, billingmill_generalledge.genledgercompanyunitaliasnumber in (‘591’,’592’) | Any form records during Fiscal Year 2024 | “%Health%Care%for%Re%entry%”, “%HCRV%”, %Veteran%Justice%Outreach%”, %VJO%”, “%Veteran%Justice%Program%”, “%VJP%”,  and not like “%VIVJOA%” | “%Health%Care%for%Re%entry%”, “%HCRV%”, %Veteran%Justice%Outreach%”, %VJO%”, “%Veteran%Justice%Program%”, “%VJP%”,  and not like “%VIVJOA%” |
| Date windows | Fiscal Year 2024 | Fiscal Year 2024 | Fiscal Year 2024 | Fiscal Year 2024 | Fiscal Year 2024 |
| Linkage keys | primarystopcodesid, secondarystopcodesid | chargeitemsid, billactivitysid | - | tiudocumentdefinitionsid, tiustandardtitlesid | eventcodevaluesid |
| Deduplication rules across sources | Unique encounter from each data source for each veteran | - | Missing form date records were excluded | - | - |

CPRS = Computerized Patient Record System. HOMES = Homeless Operations and Management Evaluation System. CDW = Corporate Data Warehouse. HCRV = Health Care for Re-entry Veterans. VJO = Veterans Justice Outreach. VJP = Veterans Justice Programs.
