# Supplementary material for: Assessing methods to identify Veterans Health Administration populations with Veterans Justice Programs contact to improve health research
Source: PLoS One. 2026 Sep 8;21(9):e0357665. doi: 10.1371/journal.pone.0357665 (PMC13552712; doi:10.1371/journal.pone.0357665)
Supplement: Supplementary file 2 [file pone.0357665.s002.docx]

**S2 Table. Specifications for opioid use disorder diagnosis and medications**

| **Measure** | **Code** | **Definition** | **Code System** |
| --- | --- | --- | --- |
| Opioid use disorder diagnosis | F11. | Opioid related disorders | ICD10CM |
|  | F11.1 | Opioid abuse | ICD10CM |
|  | F11.10 | Opioid abuse, uncomplicated | ICD10CM |
|  | F11.11 | Opioid abuse, in remission | ICD10CM |
|  | F11.12 | Opioid abuse with intoxication | ICD10CM |
|  | F11.120 | Opioid abuse with intoxication, uncomplicated | ICD10CM |
|  | F11.121 | Opioid abuse with intoxication delirium | ICD10CM |
|  | F11.122 | Opioid abuse with intoxication with perceptual disturbance | ICD10CM |
|  | F11.129 | Opioid abuse with intoxication, unspecified | ICD10CM |
|  | F11.13 | Opioid abuse with withdrawal | ICD10CM |
|  | F11.14 | Opioid abuse with opioid-induced mood disorder | ICD10CM |
|  | F11.15 | Opioid abuse with opioid-induced psychotic disorder | ICD10CM |
|  | F11.150 | Opioid abuse with opioid-induced psychotic disorder with delusions | ICD10CM |
|  | F11.151 | Opioid abuse with opioid-induced psychotic disorder with hallucinations | ICD10CM |
|  | F11.159 | Opioid abuse with opioid-induced psychotic disorder, unspecified | ICD10CM |
|  | F11.18 | Opioid abuse with other opioid-induced disorder | ICD10CM |
|  | F11.181 | Opioid abuse with opioid-induced sexual dysfunction | ICD10CM |
|  | F11.182 | Opioid abuse with opioid-induced sleep disorder | ICD10CM |
|  | F11.188 | Opioid abuse with other opioid-induced disorder | ICD10CM |
|  | F11.19 | Opioid abuse with unspecified opioid-induced disorder | ICD10CM |
|  | F11.2 | Opioid dependence | ICD10CM |
|  | F11.20 | Opioid dependence, uncomplicated | ICD10CM |
|  | F11.21 | Opioid dependence, in remission | ICD10CM |
|  | F11.22 | Opioid dependence with intoxication | ICD10CM |
|  | F11.220 | Opioid dependence with intoxication, uncomplicated | ICD10CM |
|  | F11.221 | Opioid dependence with intoxication delirium | ICD10CM |
|  | F11.222 | Opioid dependence with intoxication with perceptual disturbance | ICD10CM |
|  | F11.229 | Opioid dependence with intoxication, unspecified | ICD10CM |
|  | F11.23 | Opioid dependence with withdrawal | ICD10CM |
|  | F11.24 | Opioid dependence with opioid-induced mood disorder | ICD10CM |
|  | F11.25 | Opioid dependence with opioid-induced psychotic disorder | ICD10CM |
|  | F11.250 | Opioid dependence with opioid-induced psychotic disorder with delusions | ICD10CM |
|  | F11.251 | Opioid dependence with opioid-induced psychotic disorder with hallucinations | ICD10CM |
|  | F11.259 | Opioid dependence with opioid-induced psychotic disorder, unspecified | ICD10CM |
|  | F11.28 | Opioid dependence with other opioid-induced disorder | ICD10CM |
|  | F11.281 | Opioid dependence with opioid-induced sexual dysfunction | ICD10CM |
|  | F11.282 | Opioid dependence with opioid-induced sleep disorder | ICD10CM |
|  | F11.288 | Opioid dependence with other opioid-induced disorder | ICD10CM |
|  | F11.29 | Opioid dependence with unspecified opioid-induced disorder | ICD10CM |
| PERC STORM Model MOUD OTP HCPCS Code | G2073 | Medication assisted treatment, naltrexone; weekly bundle including dispensing and/or administration, substance use counseling, individual and group therapy, and toxicology testing if performed (provision of the services by a Medicare-enrolled opioid treatment program) | HCPCS |
|  | J2315 | Injection, naltrexone, depot form, 1 mg | HCPCS |
|  | H0020 | Alcohol and/or drug services; methadone administration and/or service (provision of the drug by a licensed program) | HCPCS |
|  | G2067 | Medication assisted treatment, methadone; weekly bundle including dispensing and/or administration, substance use counseling, individual and group therapy, and toxicology testing, if performed (provision of the services by a Medicare-enrolled opioid treatment program) | HCPCS |
|  | S0109 | Methadone, oral, 5 mg | HCPCS |
|  | G2078 | Take-home supply of methadone; up to 7 additional day supply (provision of the services by a Medicare-enrolled opioid treatment program); list separately in addition to code for primary procedure | HCPCS |
|  | J0573 | Buprenorphine/naloxone, oral, greater than 3 mg, but less than or equal to 6 mg buprenorphine | HCPCS |
|  | J0574 | Buprenorphine/naloxone, oral, greater than 6 mg, but less than or equal to 10 mg buprenorphine | HCPCS |
|  | J0575 | Buprenorphine/naloxone, oral, greater than 10 mg buprenorphine | HCPCS |
|  | J0572 | Buprenorphine/naloxone, oral, less than or equal to 3 mg buprenorphine | HCPCS |
|  | Q9991 | Injection, buprenorphine extended-release (sublocade), less than or equal to 100 mg | HCPCS |
|  | J0570 | Buprenorphine implant, 74.2 mg | HCPCS |
|  | J0571 | Buprenorphine, oral, 1 mg | HCPCS |
|  | Q9992 | Injection, buprenorphine extended-release (sublocade), greater than 100 mg | HCPCS |
|  | G2068 | Medication assisted treatment, buprenorphine (oral); weekly bundle including dispensing and/or administration, substance use counseling, individual and group therapy, and toxicology testing if performed (provision of the services by a Medicare-enrolled opioid treatment program) | HCPCS |
|  | G2070 | Medication assisted treatment, buprenorphine (implant insertion); weekly bundle including dispensing and/or administration, substance use counseling, individual and group therapy, and toxicology testing if performed (provision of the services by a Medicare-enrolled opioid treatment program) | HCPCS |
|  | G2069 | Medication assisted treatment, buprenorphine (injectable); weekly bundle including dispensing and/or administration, substance use counseling, individual and group therapy, and toxicology testing if performed (provision of the services by a Medicare-enrolled opioid treatment program) | HCPCS |
|  | G2072 | Medication assisted treatment, buprenorphine (implant insertion and removal); weekly bundle including dispensing and/or administration, substance use counseling, individual and group therapy, and toxicology testing if performed (provision of the services by a Medicare-enrolled opioid treatment program) | HCPCS |
|  | G2071 | Medication assisted treatment, buprenorphine (implant removal); weekly bundle including dispensing and/or administration, substance use counseling, individual and group therapy, and toxicology testing if performed (provision of the services by a Medicare-enrolled opioid treatment program) | HCPCS |
|  | G2079 | Take-home supply of buprenorphine (oral); up to 7 additional day supply (provision of the services by a Medicare-enrolled opioid treatment program); list separately in addition to code for primary procedure | HCPCS |
| Rx Name | Buprenorphine | Buprenorphine | Text |
|  | Naltrexone | Naltrexone | Text |
|  | Suboxone | Suboxone | Text |
|  | Subutex | Subutex | Text |

ICD10CM = International Classification of Diseases, Tenth Revision, Clinical Modification. PERC = Program and Evaluation Resource Center. STORM = Stratification Tool for Opioid Risk Mitigation. MOUD = Medications for Opioid Use Disorder. OTP = Opioid Treatment Programs. HCPCS = Healthcare Common Procedure Coding System. Rx = Prescription.
