# Supplementary material for: Assessing methods to identify Veterans Health Administration populations with Veterans Justice Programs contact to improve health research
Source: PLoS One. 2026 Sep 8;21(9):e0357665. doi: 10.1371/journal.pone.0357665 (PMC13552712; doi:10.1371/journal.pone.0357665)
Supplement: Supplementary file 3 [file pone.0357665.s003.docx]

**S3 Table. Patient and facility characteristics associated with Veterans Justice Programs identification by data source in Fiscal Year 2014**

|  |  | **Clinic stop code** |  | **HOMES record** |  | **Chart note title** |  |
| --- | --- | --- | --- | --- | --- | --- | --- |
| **Patient characteristics** | N (%) | AOR (95% CI) | *p* | AOR (95% CI) | *p* | AOR (95% CI) | *p* |
| Sex |  |  |  |  |  |  |  |
| Female | 2,480 (5%) | 1.18  (1.00, 1.39) | .039 | 0.66  (0.53, 0.81) | <.001 | 1.10  (0.90, 1.36) | .326 |
| Male | 45,013 (91%) | ref |  | ref |  | ref |  |
| Missing | 2,027 (4%) | - | - | - | - | - | - |
| Age |  |  |  |  |  |  |  |
| < 35 | 13,226 (27%) | ref |  | ref |  | ref |  |
| 35-44 | 6,774 (14%) | 0.78  (0.70, 0.87) | <.001 | 1.40  (1.18, 1.67) | <.001 | 1.07  (0.91, 1.27) | .386 |
| 45-54 | 11,506 (23%) | 0.74  (0.65, 0.83) | <.001 | 1.53  (1.25, 1.88) | <.001 | 1.06  (0.92, 1.23) | .393 |
| 55+ | 18,014 (36%) | 0.62  (0.54, 0.72) | <.001 | 1.65  (1.32, 2.06) | <.001 | 1.41  (1.16, 1.72) | .001 |
| Race/ethnicity |  |  |  |  |  |  |  |
| American  Indian/Alaskan  Native | 744 (2%) | 0.99  (0.80, 1.22) | .934 | 1.29  (0.94, 1.75) | .104 | 0.79  (0.60, 1.05) | .107 |
| Asian | 522 (1%) | 1.03  (0.72, 1.48) | .838 | 0.97  (0.62, 1.54) | .923 | 0.92  (0.57, 1.51) | .769 |
| Black | 13,779 (28%) | 1.04  (0.94, 1.16) | .418 | 0.98  (0.87, 1.11) | .794 | 0.92  (0.76, 1.12) | .438 |
| Hispanic | 3,524 (7%) | 1.09  (0.90, 1.32) | .373 | 0.75  (0.61, 0.92) | .008 | 1.10  (0.85, 1.41) | .458 |
| Multiracial | 509 (1%) | 0.86  (0.67, 1.11) | .251 | 1.30  (0.97, 1.74) | .074 | 0.95  (0.61, 1.47) | .821 |
| White | 28,413 (57%) | ref |  | ref |  | ref |  |
| Missing | 2,029 (4%) | - | - | - | - | - | - |
| Marital status |  |  |  |  |  |  |  |
| Unmarried | 36,521 (74%) | 0.89  (0.80, 0.99) | .031 | 1.33  (1.20, 1.46) | <.001 | 0.87  (0.76, 1.00) | .059 |
| Married | 9,433 (19%) | ref |  | ref |  | ref |  |
| Missing | 3,566 (7%) | - | - | - | - | - | - |
| Residence |  |  |  |  |  |  |  |
| Rural | 9,693 (20%) | 0.93  (0.79, 1.10) | .445 | 1.08  (0.89, 1.30) | .417 | 1.04  (0.78, 1.39) | .772 |
| Urban | 38,259 (77%) | ref |  | ref |  | ref |  |
| Missing | 1,568 (3%) | - | - | - | - | - | - |
| Housing status |  |  |  |  |  |  |  |
| Homeless | 22,577 (46%) | 1.03  (0.81, 1.32) | .760 | 0.71  (0.50, 1.00) | .055 | 1.45  (1.13, 1.86) | .003 |
| Housed | 26,943 (54%) | ref |  | ref |  | ref |  |
| Service-connected disability rating |  |  |  |  |  |  |  |
| None | 28,554 (58%) | ref |  | ref |  | ref |  |
| < 50% | 7,652 (16%) | 0.93  (0.86, 1.00) | .081 | 1.08  (0.98, 1.19) | .113 | 1.05  (0.93, 1.17) | .394 |
| 50-100% | 13,314 (27%) | 0.97  (0.88, 1.08) | .684 | 0.91  (0.79, 1.05) | .205 | 1.16  (1.01, 1.34) | .034 |
| Any mental health diagnosis |  |  |  |  |  |  |  |
| Yes | 30,727 (62%) | 0.98  (0.86, 1.13) | .858 | 0.98  (0.84, 1.14) | .816 | 1.10  (0.88, 1.36) | .377 |
| No | 18,793 (38%) | ref |  | ref |  | ref |  |
| Any substance use disorder diagnosis |  |  |  |  |  |  |  |
| Yes | 26,063 (53%) | 1.47  (1.32, 1.65) | <.001 | 0.66  (0.58, 0.75) | <.001 | 0.75  (0.63, 0.91) | .003 |
| No | 23,457 (47%) | ref |  | ref |  | ref |  |
| Comorbidity index, mean (SD) | 0.57 (1.35%) | 0.92  (0.90, 0.95) | <.001 | 1.05  (1.02, 1.09) | <.001 | 1.08  (1.02, 1.13) | .003 |
| **Facility characteristics** |  |  |  |  |  |  |  |
| Geographic location |  |  |  |  |  |  |  |
| Northeast | 7,080 (14%) | ref |  | ref |  | ref |  |
| South | 19,104 (39%) | 1.49  (0.88, 2.53) | .137 | 0.81  (0.40, 1.63) | .558 | 0.25  (0.04, 1.38) | .114 |
| Midwest | 11,480 (23%) | 1.36  (0.73, 2.51) | .324 | 0.75  (0.33, 1.70) | .499 | 0.41  (0.05, 2.98) | .384 |
| West | 11,856 (24%) | 1.60  (0.87, 2.96) | .129 | 0.71 (0.32, 1.58) | .408 | 0.23  (0.03, 1.76) | .161 |
| Location |  |  |  |  |  |  |  |
| Rural | 12,950 (26%) | 0.79  (0.50, 1.25) | .327 | 0.75  (0.39, 1.46) | .406 | 4.68  (1.27, 17.10) | .020 |
| Urban | 36,570 (74%) | ref |  | ref |  | ref |  |

*Note*. Missing values were not included in the models. HOMES = Homeless Operations and Management Evaluation System. AOR = adjusted odds ratio. CI = confidence interval. Ref = reference group. SD = standard deviation.
