# Supplementary material for: Assessing methods to identify Veterans Health Administration populations with Veterans Justice Programs contact to improve health research
Source: PLoS One. 2026 Sep 8;21(9):e0357665. doi: 10.1371/journal.pone.0357665 (PMC13552712; doi:10.1371/journal.pone.0357665)
Supplement: Supplementary file 4 [file pone.0357665.s004.docx]

**S4 Table. Patient and facility characteristics associated with Veterans Justice Programs identification by data source in Fiscal Year 2019**

|  |  | **Clinic stop code** |  | **HOMES record** |  | **Chart note title** |  |
| --- | --- | --- | --- | --- | --- | --- | --- |
| **Patient characteristics** | N (%) | AOR (95% CI) | *p* | AOR (95% CI) | *p* | AOR (95% CI) | *p* |
| Sex |  |  |  |  |  |  |  |
| Female | 3,501 (6%) | 1.04  (0.90, 1.21) | .552 | 0.84  (0.67, 1.06) | .155 | 1.03  (0.84, 1.27) | .752 |
| Male | 49,071 (89%) | ref |  | ref |  | ref |  |
| Missing | 2,337 (4%) | - | - | - | - | - | - |
| Age |  |  |  |  |  |  |  |
| < 35 | 13,412 (24%) | ref |  | ref |  | ref |  |
| 35-44 | 11,707 (21%) | 0.93  (0.84, 1.03) | .191 | 1.09  (0.94, 1.26) | .225 | 1.05  (0.91, 1.20) | .476 |
| 45-54 | 9,175 (17%) | 0.94  (0.81, 1.08) | .403 | 1.05  (0.88, 1.25) | .572 | 1.06  (0.88, 1.29) | .499 |
| 55+ | 20,615 (38%) | 0.82  (0.73, 0.92) | .001 | 1.26  (1.09, 1.47) | .002 | 1.16  (1.01, 1.34) | .034 |
| Race/ethnicity |  |  |  |  |  |  |  |
| American  Indian/Alaskan  Native | 740 (1%) | 1.09  (0.83, 1.44) | .514 | 0.81  (0.57, 1.17) | .275 | 1.02  (0.65, 1.60) | .922 |
| Asian | 787 (1%) | 1.09  (0.84, 1.43) | .490 | 0.78  (0.49, 1.22) | .283 | 1.02  (0.73, 1.42) | .870 |
| Black | 13,848 (25%) | 1.12  (1.02, 1.24) | .016 | 1.03  (0.90, 1.17) | .631 | 0.79  (0.70, 0.91) | .001 |
| Hispanic | 4,808 (9%) | 0.96  (0.83, 1.10) | .575 | 1.01  (0.83, 1.23) | .872 | 1.06  (0.89, 1.27) | .468 |
| Multiracial | 589 (1%) | 1.08  (0.82, 1.43) | .545 | 0.82  (0.52, 1.30) | .404 | 0.98  (0.70, 1.39) | .951 |
| White | 31,793 (58%) | ref |  | ref |  | ref |  |
| Missing | 2,344 (4%) | - | - | - | - | - | - |
| Marital status |  |  |  |  |  |  |  |
| Unmarried | 39,031 (71%) | 0.89  (0.82, 0.96) | .005 | 1.25  (1.09, 1.44) | .001 | 1.02  (0.93, 1.12) | .610 |
| Married | 13,299 (24%) | ref |  | ref |  | ref |  |
| Missing | 2,579 (5%) | - | - | - | - | - | - |
| Residence |  |  |  |  |  |  |  |
| Rural | 11,054 (20%) | 0.97  (0.86, 1.09) | .659 | 0.82  (0.70, 0.96) | .014 | 1.17  (1.01, 1.35) | .031 |
| Urban | 42,926 (78%) | ref |  | ref |  | ref |  |
| Missing | 929 (2%) | - | - | - | - | - | - |
| Housing status |  |  |  |  |  |  |  |
| Homeless | 21,168 (39%) | 1.05  (0.91, 1.22) | .448 | 0.76  (0.63, 0.92) | .007 | 1.08  (0.89, 1.31) | .424 |
| Housed | 33,741 (61%) | ref |  | ref |  | ref |  |
| Service-connected disability rating |  |  |  |  |  |  |  |
| None | 23,656 (43%) | ref |  | ref |  | ref |  |
| < 50% | 8,519 (16%) | 0.94  (0.86, 1.04) | .246 | 1.05  (0.90, 1.22) | .500 | 1.06  (0.93, 1.20) | .344 |
| 50-100% | 22,734 (41%) | 0.88  (0.80, 0.96) | .009 | 1.09  (0.96, 1.24) | .167 | 1.14  (1.00, 1.30) | .040 |
| Any mental health diagnosis |  |  |  |  |  |  |  |
| Yes | 36,497 (66%) | 0.85  (0.75, 0.95) | .007 | 1.12  (0.97, 1.30) | .112 | 1.20  (1.03, 1.40) | .018 |
| No | 18,412 (34%) | ref |  | ref |  | ref |  |
| Any substance use disorder diagnosis |  |  |  |  |  |  |  |
| Yes | 28,244 (51%) | 1.40  (1.24, 1.58) | <.001 | 0.60  (0.52, 0.69) | <.001 | 0.81  (0.70, 0.95) | .009 |
| No | 26,665 (49%) | ref |  | ref |  | ref |  |
| Comorbidity index, mean (SD) | 0.70 (1.53) | 0.94  (0.91, 0.97) | <.001 | 1.03  (0.99, 1.08) | .127 | 1.07  (1.03, 1.10) | <.001 |
| **Facility characteristics** |  |  |  |  |  |  |  |
| Geographic location |  |  |  |  |  |  |  |
| Northeast | 7,275 (13%) | ref |  | ref |  | ref |  |
| South | 22,114 (40%) | 1.33  (0.67, 2.66) | .412 | 0.68  (0.33, 1.41) | .307 | 0.74  (0.18, 3.03) | .682 |
| Midwest | 12,090 (22%) | 0.72  (0.34, 1.51) | .390 | 1.67  (0.74, 3.75) | .213 | 0.85  (0.17, 4.29) | .853 |
| West | 13,430 (24%) | 0.77  (0.36, 1.64) | .512 | 1.41  (0.64, 3.08) | .390 | 1.04  (0.21, 5.14) | .960 |
| Location |  |  |  |  |  |  |  |
| Rural | 12,167 (22%) | 0.86  (0.52, 1.43) | .577 | 0.97  (0.54, 1.72) | .927 | 2.04  (0.72, 5.73) | .176 |
| Urban | 42,742 (78%) | ref |  | ref |  | ref |  |

*Note*. Missing values were not included in the models. HOMES = Homeless Operations and Management Evaluation System. AOR = adjusted odds ratio. CI = confidence interval. Ref = reference group. SD = standard deviation.
