# Supplementary material for: Assessing methods to identify Veterans Health Administration populations with Veterans Justice Programs contact to improve health research
Source: PLoS One. 2026 Sep 8;21(9):e0357665. doi: 10.1371/journal.pone.0357665 (PMC13552712; doi:10.1371/journal.pone.0357665)
Supplement: Supplementary file 5 [file pone.0357665.s005.docx]

Dear Dr. Cioffi,

We thank you and the reviewers for the thoughtful comments on our manuscript entitled, “Assessing methods to identify Veteran Health Administration populations with Veterans Justice Programs contact to improve health research” (PONE-D-25-62496). We greatly appreciate your insightful feedback and have no doubt our manuscript is strengthened as a result. Below are the comments followed by our responses.

**Editor**

**Comment #1:** Thank you for submitting your manuscript to PLOS ONE. After careful consideration, we feel that it has merit but does not fully meet PLOS ONE’s publication criteria as it currently stands. Therefore, we invite you to submit a revised version of the manuscript that addresses the points raised during the review process.

**Response:** Thank you!

**Comment #2:** The most significant issue concerns the methodological validation of the proposed identification strategy. The manuscript convincingly demonstrates that reliance on any single data source would lead to incomplete ascertainment of veterans with Veterans Justice Programs (VJP) contact. However, the current analysis remains largely descriptive and does not provide a formal validation of the combined approach. In the absence of a gold standard, the recommendation to use all three data sources is reasonable but presently insufficiently substantiated. The manuscript would be strengthened by additional analyses that assess concordance between sources, quantify agreement, and estimate potential misclassification. Where feasible, validation using chart review, linkage to external justice system data, or sensitivity analyses exploring alternative definitions would substantially improve the methodological rigor. If a gold standard is not available, this limitation should be more fully explored and the conclusions framed more cautiously.

**Response:** To assess concordance between sources, we included Fleiss’ kappa tests for VJP, HCRV, and VJO. To examine validation, we tested patient and facility factors associated with each type of data source using the overall VJP sample. Results indicated that patient factors varied in their association with each type of data source. For example, female veterans had lower odds of having a HOMES records and higher odds of having a chart note compared to male veterans. Given that there is no gold standard available, we expanded our limitations to more fully explore this point and framed our conclusions more cautiously.

**Comment #3:** A related concern is the limited statistical depth of the analysis. The current presentation focuses primarily on counts, percentages, and descriptive overlap across data sources. For a methodological study, readers would benefit from more formal measures of agreement and reliability, such as kappa statistics or other concordance metrics, as well as sensitivity analyses examining how population characteristics or program-level factors influence identification across sources. Additional analyses clarifying the implications of using one versus multiple sources for downstream research would also enhance the contribution of the paper.

**Response:** Thank you for these suggestions. As described in our response to Comment #2, we added Fleiss’ kappa tests, examined the association between patient and facility factors and different data sources. We also explored variation in the prevalence of opioid use disorder diagnosis and receipt of medications for opioid use disorder depending on the data source used. We also calculated the prevalence of a clinical condition (i.e., opioid use disorder) and a quality measure for this clinical condition (i.e., receipt of medications for opioid use disorder). Depending on the data source used, the prevalence of opioid use disorder ranged from 5.3% for *clinic code only* group to 13.8% for the *all three data sources* group (an 8.5% range). Receipt of medications for opioid use disorder ranged from 51.3% for the *clinic codes + HOMES* group to 57.1% for the *HOMES + chart note* group (a 5.8% range). Clinic conditions and performance measures are monitored at the VHA; thus, it is important to use the most accurate approach to identifying the population of interest to ensure that other measures based on this population sample are correctly calculated and reported.

**Comment #4:** The discussion section would also benefit from further refinement. At present, the conclusions occasionally extend beyond what can be supported by the data, particularly in recommending the universal use of all three data sources. These recommendations should be framed more cautiously and clearly situated within the limitations of the study design. A more explicit discussion of generalizability—both within the VHA and in relation to other healthcare systems—would also strengthen the manuscript.

**Response:** We extensively revised the Discussion section to frame our results more cautiously and describe the generalizability of the results.

**Comment #5:** Finally, several technical and reporting issues should be addressed in revision. The data availability statement should be clarified to ensure full compliance with journal policy.

**Response:** We have updated the data availability statement.

**Comment #6:** Some sections of the discussion repeat points made earlier and could be streamlined for clarity.

**Response:** We revised and streamlined the discussion section.

**Comment #7:** Figures illustrating overlap across data sources are useful but would benefit from additional interpretation within the text.

**Response:** Additional information on how to interpret the figure was added to the Results.

**Comment #8:** The exploratory analysis of ICD-10 codes is interesting but currently underdeveloped; either expanding its analytic contribution or more clearly framing it as preliminary would improve coherence.

**Response:** Given that we added new analyses, we decided to remove the exploratory ICD-10 codes and focus our analyses on the data sources.

**Comment #9:** In summary, we believe this manuscript addresses an important methodological challenge and has the potential to make a meaningful contribution. However, substantial revision is required to strengthen the validation strategy, expand the analytic depth, and align the conclusions more closely with the evidence presented. We would welcome a carefully revised version that addresses these and reviewers' concerns.

**Response:** Thank you – we look forward to seeing your comments on our revised manuscript.

**Comment #10:** When submitting your revision, we need you to address these additional requirements.

1. Please ensure that your manuscript meets PLOS ONE's style requirements, including those for file naming. The PLOS ONE style templates can be found at

<https://journals.plos.org/plosone/s/file?id=wjVg/PLOSOne_formatting_sample_main_body.pdf> and

[https://journals.plos.org/plosone/s/file?id=ba62/PLOSOne_formatting_sample_title_authors_affiliations.pdf](https://gcc02.safelinks.protection.outlook.com/?url=https%3A%2F%2Ftrack.editorialmanager.com%2FCL0%2Fhttps%3A%252F%252Fjournals.plos.org%252Fplosone%252Fs%252Ffile%253Fid%3Dba62%252FPLOSOne_formatting_sample_title_authors_affiliations.pdf%2F1%2F010f019c929487b5-1322c137-97eb-4c88-809d-eccd4c0c1be8-000000%2FhgIDhhX2dXs8VXlOQDoNFgSNmIG4YDKHKkjcO41AAWA%3D251&data=05%7C02%7C%7C1b75c4c3582047ffdc4708de7413c528%7Ce95f1b23abaf45ee821db7ab251ab3bf%7C0%7C0%7C639075825765843775%7CUnknown%7CTWFpbGZsb3d8eyJFbXB0eU1hcGkiOnRydWUsIlYiOiIwLjAuMDAwMCIsIlAiOiJXaW4zMiIsIkFOIjoiTWFpbCIsIldUIjoyfQ%3D%3D%7C0%7C%7C%7C&sdata=mebdokrILmBkvpQAFvJ0ZElCGN83oG8Ia9%2FDoIZZzVY%3D&reserved=0).

**Response:** We aligned and double checked our revisions to meet PLOS ONE’s style requirements.

**Comment #11:** 2. Thank you for stating in your Funding Statement:

“Research reported in this publication was supported by the Department of Veterans Affairs Health Systems Research (Dr Finlay, VA HSR IIR 20–040). Dr. Sox-Harris was funded by a grant from the Department of Veterans Affairs Health Systems Research (RCS 14-232).

The views expressed in this article are those of the authors and do not necessarily reflect the position or policy of the Department of Veterans Affairs or the United States government. The funding organization had no role in the study design; analysis and interpretation of data; in the writing of the report; or in the decision to submit the paper for publication.

Funder URL: <https://www.hsrd.research.va.gov/>”

Please provide an amended statement that declares *all* the funding or sources of support (whether external or internal to your organization) received during this study, as detailed online in our guide for authors at <http://journals.plos.org/plosone/s/submit-now>.  Please also include the statement “There was no additional external funding received for this study.” in your updated Funding Statement.

Please include your amended Funding Statement within your cover letter. We will change the online submission form on your behalf.

**Response:** The amended Funding Statement is included in the cover letter.

**Comment #12:** 3. In the online submission form, you indicated that your data is available only on request from a third party. Please note that your Data Availability Statement is currently missing [the name of the third party contact or institution / contact details for the third party, such as an email address or a link to where data requests can be made]. Please update your statement with the missing information.

**Response:** The Data Availability Statement has been revised to include this information.

**Comment #13:** 4. If the reviewer comments include a recommendation to cite specific previously published works, please review and evaluate these publications to determine whether they are relevant and should be cited. There is no requirement to cite these works unless the editor has indicated otherwise.

**Response:** No specific previously published works were recommended by the reviewers.

**Reviewer #1**

This manuscript describes methodology for accurately capturing Veterans eligible for care through VHA that have criminal legal involvement. The authors note that three administrative/clinical databases may capture this legal involvement, but for various reasons they are each incomplete. Identifying the "completeness" of each database compared with the overall merged dataset (there is no "gold standard" for comparison) helps standardize populations for studies--improving overall quality, reliability, and reproducibility.

**Response:** Thank you!

**Reviewer #2**

Thank you for the opportunity to review this manuscript is best read as a methodological, descriptive study (not a traditional narrative “review”) that examines how to identify Veterans Health Administration (VHA) patients with contact with Veterans Justice Programs (VJP) using routinely available electronic health record/administrative data.

The topic is important, and the paper highlights a real problem: different data sources identify different groups of veterans, which can affect research and quality improvement efforts.

**Response:** Thank you!

Several important issues should be noted.
Major comments:
**Comment #1:** Without a reference standard, the work cannot quantify sensitivity/specificity or false positives, so the core conclusion (“use all three”) is reasonable but not definitively proven as “most accurate.” This limits scientific impact because it remains unclear how close the combined method is to “truth.”

**Response:** We have expanded upon this limitation and noted that it may not be feasible to compared these data with data from criminal legal sources because of the number of potential sources (i.e., law enforcement for arrests, courts, jails, prisons, and community supervision departments). We have also tempered our conclusions to reflect this limitation.

**Comment #2:** The study convincingly shows disagreement across sources, but does not identify which veterans are missed by each method or whether missedness is patterned by site, setting (court vs jail), or program workflow—issues that directly affect downstream bias in etiologic or quality-of-care studies.

**Response:** We added additional analyses to examine the association between patient and facility factors and different data sources, and explored variation in the prevalence of opioid use disorder diagnosis and receipt of medications for opioid use disorder depending on the data source used to shed light on differences that would be observed depending on the data sources used.


**Comment #3:** Because documentation and EHR environments evolve, a one-year analysis may not generalize across time or modernization phases; this matters if the paper’s goal is to offer a durable standard definition for ongoing research.
**Response:** We included two additional Fiscal Years of data, 2014 and 2019, and examined changes in data sources over time.


**Comment #4:** Excluding veterans with VJP contact who are ineligible for VHA care narrows the population and may obscure important policy-relevant groups (e.g., those falling through coverage gaps), depending on the intended use of the algorithm.

**Response:** We now note in the Methods section that we excluded 929 veterans in Fiscal Year 2024 because they were ineligible for VHA care or who did not have CDW electronic health records because these veterans did not have the patient information necessary for our exploration of patient factors associated with different data sources and examination of diagnosis and receipt of medications for opioid use disorder. We also added this limitation to Discussion section.

**Comment #5:** The paper’s direct applicability is largely confined to VHA/VJP; for journals with broader readership, this reduces perceived contribution unless paired with a stronger methodological template transferable to other systems.
**Response:** We agree that the direct applicability is largely focused on VHA and have added this as a limitation.


**Comment #6:** I recommend major revision, and I encourage you to continue this important line of work.

**Response:** Thank you!

**Reviewer #3:**
I have reviewed your manuscript, “Assessing Methods to Identify Veteran Health Administration Populations with Veterans Justice Programs Contact to Improve Health Research.” Overall, this is a practically important methods paper for VHA researchers and operational teams. The core idea, demonstrating that reliance on a single data source materially under-identifies VJP contact, is compelling and well aligned with PLOS ONE’s emphasis on methodological rigor and reproducibility. However, I recommend major revisions before the work is ready for publication, mainly to (i) resolve internal inconsistencies in counts, (ii) strengthen reproducibility of the cohort-identification algorithm, and (iii) address PLOS ONE expectations for transparent “data underlying the results” given restricted VA data.

**Response:** Thank you – we have resolved internal inconsistencies, strengthened the reproducibility of the cohort-identification algorithm, and provided additional information about data access. Details are described below. For example, we now include S1 Appendix with specifications for the algorithm. We also updated the Data Availability Statement to include contact information to request data access.

Abstract
**Comment #1**: Fix all sample-size inconsistencies and align with the cohort flow (Fig 1) and Results text. The abstract reports 52,548 VHA-eligible veterans, while the Results/flow indicate Any VJP contact N=53,617 and Ineligible excluded N=950, which do not reconcile arithmetically. Ensure the same numbers appear in Abstract, Results, and figures, and explicitly state any additional exclusions if applicable.

**Response:** We triple-checked all numbers to ensure they align throughout the documents.

**Comment #2:** Define the unit of “contact” and “identified through.” Clarify whether a “contact” is an encounter, a note/form record, a unique day, or some other operational unit, especially since you report “average number of 5.3 contacts (range 1–120)” without defining the metric.

**Response:** We added the definition of contact. We removed the term “identified through” to improve clarity. We removed the average contacts from the Results section for simplicity as our manuscript is not focused on multiple contacts, but rather on identifying veterans who have any contact with VJP.

Introduction
**Comment #**3: Clearly distinguish “criminal legal involvement” from “VJP contact.” Frame the primary outcome as VJP contact (an ascertainable EHR construct) and acknowledge that it is an imperfect proxy for broader criminal legal involvement. This prevents over-claiming and matches what the data actually means.

**Response:** We revised throughout the manuscript to clarify that we were identifying VJP contact and that it is an imperfect proxy for broader criminal legal involvement.

**Comment #4:** Strengthen the rationale for the three-source approach with expected bias mechanisms. You already explain why HOMES may be incomplete; add parallel expectations for: Stop codes (coding variability, missed/incorrect workload documentation); Note titles (facility-specific naming conventions, EHR modernization effects)
**Response:** Thank you for these suggestions. We added them to Introduction.

**Comment #5:** Consider tighten study aims into 1–2 explicit objectives. For example: (1) quantify overlap and under-ascertainment when using single vs multi-source definitions; (2) provide a reproducible cohort-identification specification usable in CDW/VINCI contexts.

**Response:** We updated our objectives as suggested.


Methodology
**Comment #6:** Make the identification algorithm reproducible (publishable specification). For each source, report: exact CDW tables/fields, inclusion filters, date windows, and linkage keys; define deduplication rules across sources.

**Response:** We added S1 Appendix to include this information in the manuscript.

**Comment #7:** Stop codes: specify data origin and operationalization. State which workload/encounter tables were queried, how stop codes were applied, and whether multiple encounters per person were counted (and how).

**Response:** Please see S1 Appendix for additional information on the stop codes, workload tables, and other related information.

**Comment #8:** HOMES: specify the exact “entry/exit” definition and data handling. Identify the forms/fields used, how multiple records were handled, and how veterans were classified when entry/exit dates were missing.

**Response:** Please see S1 Appendix for additional information on HOMES specifications.

**Comment #9:** Define “contact” operationally and specify how you computed “average contacts.” If combining heterogeneous records (encounters + forms + notes), define whether “contacts” are raw records, unique days, or events.

**Response:** We added the definition of contact for VJP, HCRV, and VJO to the Measures section, including whether it was an encounter, form, or chart note title. We removed the average contacts from the Results section for simplicity as our manuscript is not focused on multiple contacts, but rather on identifying veterans who have any contact with VJP.

**Comment #10:** Clarify EHR modernization considerations. If you list Millennium sites, state whether you analyzed differences by system (even descriptively). If not, specify this clearly as a limitation and discuss likely direction of bias.

**Response:** We now include as a limitation that Millennium data was included in our study, but we did not analyze differences by system.

**Comment #11:** ICD-10 comparison: correct code inconsistencies and define the time window. Table 1 uses Y92.14x, while the Results text refers to “Y92.4” (likely a typo). Also clarify whether ICD-10 codes were searched only in FY2024 or historically.

**Response:** We removed the ICD-10 comparisons and focused our analyses on examining the data sources that identify VJP contacts.

**Comment #12:** Data Availability (PLOS ONE): provide a concrete access pathway + share underlying aggregates and code lists. Even if individual-level VA data cannot be public, provide: how qualified researchers can request access (governance route), and aggregated counts underlying tables/figures + full code lists/queries as Supporting Information.

**Response:** We updated the Data Availability and added Supporting Information about the data sources and codes used.

Results
**Comment #13:** Reconcile all denominators across text, tables, and figures. The “Any VJP contact / ineligible excluded / eligible” counts currently do not reconcile. The HCRV sample size differs between text/Table 2 (n=8.541) and Fig 3a (n=8.556). Fix these before resubmission; inconsistent denominators are a major credibility issue.

**Response:** We triple-checked all numbers throughout the paper to ensure consistency.

**Comment #14:** Add a simple table quantifying “single-source capture” and “two-source capture.” Venn diagrams are helpful, but readers need a clear numeric summary of how many veterans would be missed if using: stop codes only; HOMES only; note titles only; each pairwise combination. This directly supports your core recommendation (“use all three sources”).

**Response:** We added Table 2 to provide a clear numeric summary of veterans who are identified using each data source and combinations of data sources.

**Comment #15:** Ensure subgroup reporting (HCRV and VJO) is consistent with figures. Verify overlap percentages and Ns for HCRV/VJO across all outputs; align text statements with Fig 3a/3b.
**Response:** We triple-checked subgroup reporting and all numbers throughout the paper to ensure consistency.

Discussion
**Comment #16:** Temper over-strong claims about imputation and missing data. Instead of asserting imputation would be “unreliable,” specify that high missingness and likely non-random mechanisms may limit correction; emphasize that HOMES alone is insufficient without multi-source augmentation.

**Response:** The Discussion was substantially revised and no longer includes information on imputation and missing data.

**Comment #17:** Provide practical guardrails to minimize stigmatization risk. You mention discrimination concerns; add 1–2 concrete implementation principles (role-based access, purpose limitation) to align operational use with ethical practice.

**Response:** We appreciate these suggestions and added them to the Discussion.

**Comment #18:** Briefly reinforce transparency commitments. State that code lists and aggregate data underlying figures/tables are provided in Supplementary files (once added), consistent with PLOS ONE transparency expectations.

**Response:** We note in the Discussion that we include information about code lists in the Supporting Information.

Conclusion
**Comment #19:** Make the conclusion explicitly methodological and avoid overgeneralizing to “criminal legal involvement.” Use “veterans with VJP contact” as the measured construct.

**Response:** We revised the conclusion as suggested.

**Comment #20:** State the actionable recommendation and the deliverable. Example structure: Single-source ascertainment materially under-identifies VJP contact. A standardized multi-source definition is recommended.

**Response:** We revised the conclusion as suggested.

**Comment 21:** Overall
Your manuscript addresses an important VHA methods problem, but it needs key fixes before it can be re-evaluated: (1) reconcile all sample-size/denominator inconsistencies across the Abstract, Results, and Fig 1; (2) define the operational unit of “contact” and “identified through” (and how contacts are counted/merged across stop codes, HOMES, and notes); and (3) consistently frame the primary outcome as “VJP contact” (an EHR-ascertainable construct) rather than broader “criminal legal involvement,” which is only a proxy. Therefore I recommended major revision.

**Response:** Please see above for specifics of how these comments were addressed.

To ensure your figures meet our technical requirements, please review our figure guidelines: <https://journals.plos.org/plosone/s/figures>

You may also use PLOS’s free figure tool, NAAS, to help you prepare publication quality figures: <https://journals.plos.org/plosone/s/figures#loc-tools-for-figure-preparation>.

NAAS will assess whether your figures meet our technical requirements by comparing each figure against our figure specifications.

**Response:** We reviewed the figure guidelines and used the NAAS tool to check that our figures met the technical requirements.

We appreciate the opportunity to revise our manuscript.

Thank you,

The Authors
