# Supplementary material for: Assessing methods to identify Veterans Health Administration populations with Veterans Justice Programs contact to improve health research
Source: PLoS One. 2026 Sep 8;21(9):e0357665. doi: 10.1371/journal.pone.0357665 (PMC13552712; doi:10.1371/journal.pone.0357665)
Supplement: Supplementary file 6 [file pone.0357665.s006.docx]

Dear Dr. Cioffi,

We thank you and the reviewers for the thoughtful comments on our revised manuscript entitled, “Assessing methods to identify Veteran Health Administration populations with Veterans Justice Programs contact to improve health research” (PONE-D-25-62496R1). We greatly appreciate your insightful feedback and have no doubt our manuscript is strengthened as a result. Below are the comments followed by our responses.

**Editor**

**Journal Requirements:**

**Comment #1:** If the reviewer comments include a recommendation to cite specific previously published works, please review and evaluate these publications to determine whether they are relevant and should be cited. There is no requirement to cite these works unless the editor has indicated otherwise.

**Response:** There were no recommendations from reviewers to cite specific previously published works.

**Comment #2:** Please review your reference list to ensure that it is complete and correct. If you have cited papers that have been retracted, please include the rationale for doing so in the manuscript text, or remove these references and replace them with relevant current references. Any changes to the reference list should be mentioned in the rebuttal letter that accompanies your revised manuscript. If you need to cite a retracted article, indicate the article’s retracted status in the References list and also include a citation and full reference for the retraction notice.

**Response:** We reviewed the reference list to ensure it was complete and did not identify any cited papers that were retracted. We updated one reference, replacing the Deyo comorbidity index with an updated version of the Charlson comorbidity index from 2005. We also made minor modifications to some references (e.g., changing a full journal name to the journal abbreviation).

**Additional Editor Comments:**

**Comment #3:** Although the manuscript is now close to being suitable for publication, I believe that a few final revisions would further strengthen the presentation. First, while the conclusions have been moderated appropriately compared with the previous version, I encourage the authors to maintain consistently cautious language when recommending the combined use of all three data sources. Given the absence of an external reference standard, it cannot be definitively established that the combined approach represents the most accurate method of identifying veterans with VJP contact. Rather, the evidence supports the conclusion that, among the approaches evaluated in this study, the combined strategy provides the most comprehensive ascertainment. Maintaining this distinction throughout the Discussion and Conclusions will ensure that the recommendations remain fully aligned with the available evidence.

**Response:** We updated the Abstract, Discussion, and Conclusions to temper our recommendations for the use of all three data sources to ensure we are aligned with the available evidence.

**Comment #4:** Second, although the limitations section has been substantially expanded, a brief additional emphasis on the implications of the absence of a true gold standard would be valuable. Specifically, it would be helpful to reiterate that the study evaluates concordance among existing data sources rather than diagnostic accuracy, and that future validation against external justice-system data, where feasible, would further strengthen confidence in the proposed identification strategy.
**Response:** We have added this additional emphasis to the Limitations section to reiterate the focus on evaluating concordance among existing data sources rather than diagnostic accuracy. We also revised the following sentence “future research…” to “future validation against external criminal legal system data, where feasible, would further strengthen confidence in the proposed identification strategy.”

**Comment #5:** Finally, I encourage the authors to perform one final careful review of the manuscript to ensure complete consistency of terminology, numerical values, and descriptions across the Abstract, Results, tables, figures, and Supporting Information. The revised manuscript appears considerably improved in this regard, and a final editorial check will help ensure complete consistency throughout.
**Response:** We had two authors conduct a complete check of the manuscript and appendices to ensure consistency throughout.

**Reviewer #2**

**Initial Comment:** Thank you for providing the revised manuscript and responses to the comments. I have reviewed the revision and have no further questions or comments.

**Response:** Thank you!

**Reviewer #3**

**Initial Comment:** Thank you for the revised version of the manuscript. The manuscript has been substantially improved, and the main methodological concerns raised in the previous review have largely been addressed. In particular, the cohort counts are now much clearer, the primary construct is better framed as VJP contact, the operational definition of contact has been added, and Table 2 provides a useful summary of single-source and combined-source identification patterns. I recommend only minor revisions before publication.

**Response:** Thank you!

**Comment #1:** Align percentages across the Abstract, Results, and tables. The Abstract reports 81% clinic stop codes and 64% chart note titles, whereas the Results section/ under Table 2 (row 230 and 231), reports 80% and 63%, respectively. Please harmonize these values throughout the manuscript.

**Response:** We had two authors conduct a complete check of the manuscript and appendices to ensure consistency throughout and harmonized any mismatched values.

**Comment #2:** Further refine the framing of VJP contact vs. criminal legal involvement. The manuscript is now much clearer, but one sentence in the Introduction still states that the focus is on veterans with criminal legal involvement (row 82-86). Please consider revising this to emphasize that the study focuses on veterans with VJP contact, used as a proxy for broader criminal legal involvement.

**Response:** We clarified in the Introduction that this study was limited to veterans who received VJP outreach services from HCRV and VJO staff. The following sentence now notes that researchers have used encounters with these programs as a broad proxy for criminal legal involvement.

**Comment #3:** Correct minor typographical and abbreviation issues. For example, Table 2 includes “Fiscsl Year 2024,” and the table note appears to define VJP twice, including one instance where “VJP = Veterans Justice Outreach” should likely be “VJO = Veterans Justice Outreach.” Overall, the revised manuscript is much stronger and now provides a clearer and more useful methodological contribution. After these minor corrections, I believe the manuscript will be suitable for publication.

**Response:** We corrected the minor typographical and abbreviations noted, including changing “Fiscsl” to “Fiscal” and “VJP” to “VJO” in Table 2. We also had two authors check the manuscript closely for any additional typos.

We appreciate the opportunity to revise our manuscript.

Thank you,

The Authors
