# Supplementary material for: Assessing methods to identify Veterans Health Administration populations with Veterans Justice Programs contact to improve health research
Source: PLoS One. 2026 Sep 8;21(9):e0357665. doi: 10.1371/journal.pone.0357665 (PMC13552712; doi:10.1371/journal.pone.0357665)
Supplement: Peer review history

# Peer review: Decision Letter 0

**DOI:** 10.1371/journal.pone.0357665.r001

**Authors:** Andrea Cioffi

24 Feb 2026

Dear Dr. Finlay,

Thank you for submitting your manuscript to PLOS ONE. After careful consideration, we feel that it has merit but does not fully meet PLOS ONE’s publication criteria as it currently stands. Therefore, we invite you to submit a revised version of the manuscript that addresses the points raised during the review process.

The most significant issue concerns the methodological validation of the proposed identification strategy. The manuscript convincingly demonstrates that reliance on any single data source would lead to incomplete ascertainment of veterans with Veterans Justice Programs (VJP) contact. However, the current analysis remains largely descriptive and does not provide a formal validation of the combined approach. In the absence of a gold standard, the recommendation to use all three data sources is reasonable but presently insufficiently substantiated. The manuscript would be strengthened by additional analyses that assess concordance between sources, quantify agreement, and estimate potential misclassification. Where feasible, validation using chart review, linkage to external justice system data, or sensitivity analyses exploring alternative definitions would substantially improve the methodological rigor. If a gold standard is not available, this limitation should be more fully explored and the conclusions framed more cautiously.

A related concern is the limited statistical depth of the analysis. The current presentation focuses primarily on counts, percentages, and descriptive overlap across data sources. For a methodological study, readers would benefit from more formal measures of agreement and reliability, such as kappa statistics or other concordance metrics, as well as sensitivity analyses examining how population characteristics or program-level factors influence identification across sources. Additional analyses clarifying the implications of using one versus multiple sources for downstream research would also enhance the contribution of the paper.

The discussion section would also benefit from further refinement. At present, the conclusions occasionally extend beyond what can be supported by the data, particularly in recommending the universal use of all three data sources. These recommendations should be framed more cautiously and clearly situated within the limitations of the study design. A more explicit discussion of generalizability—both within the VHA and in relation to other healthcare systems—would also strengthen the manuscript.

Finally, several technical and reporting issues should be addressed in revision. The data availability statement should be clarified to ensure full compliance with journal policy. Some sections of the discussion repeat points made earlier and could be streamlined for clarity. Figures illustrating overlap across data sources are useful but would benefit from additional interpretation within the text. The exploratory analysis of ICD-10 codes is interesting but currently underdeveloped; either expanding its analytic contribution or more clearly framing it as preliminary would improve coherence.

In summary, we believe this manuscript addresses an important methodological challenge and has the potential to make a meaningful contribution. However, substantial revision is required to strengthen the validation strategy, expand the analytic depth, and align the conclusions more closely with the evidence presented. We would welcome a carefully revised version that addresses these and reviewers' concerns.

Please submit your revised manuscript by Apr 10 2026 11:59PM. If you will need more time than this to complete your revisions, please reply to this message or contact the journal office at plosone@plos.org. When you're ready to submit your revision, log on to https://www.editorialmanager.com/pone/ and select the 'Submissions Needing Revision' folder to locate your manuscript file.

- A letter that responds to each point raised by the academic editor and reviewer(s). You should upload this letter as a separate file labeled 'Response to Reviewers'.
- A marked-up copy of your manuscript that highlights changes made to the original version. You should upload this as a separate file labeled 'Revised Manuscript with Track Changes'.
- An unmarked version of your revised paper without tracked changes. You should upload this as a separate file labeled 'Manuscript'.

If you would like to make changes to your financial disclosure, please include your updated statement in your cover letter. Guidelines for resubmitting your figure files are available below the reviewer comments at the end of this letter.

If applicable, we recommend that you deposit your laboratory protocols in protocols.io to enhance the reproducibility of your results. Protocols.io assigns your protocol its own identifier (DOI) so that it can be cited independently in the future. For instructions see: https://journals.plos.org/plosone/s/submission-guidelines#loc-laboratory-protocols. Additionally, PLOS ONE offers an option for publishing peer-reviewed Lab Protocol articles, which describe protocols hosted on protocols.io. Read more information on sharing protocols at https://plos.org/protocols?utm_medium=editorial-email&utm_source=authorletters&utm_campaign=protocols.

We look forward to receiving your revised manuscript.

Kind regards,

Andrea Cioffi

Academic Editor

PLOS One

Journal Requirements:

When submitting your revision, we need you to address these additional requirements.

1. Please ensure that your manuscript meets PLOS ONE's style requirements, including those for file naming. The PLOS ONE style templates can be found at

https://journals.plos.org/plosone/s/file?id=wjVg/PLOSOne_formatting_sample_main_body.pdf and

https://journals.plos.org/plosone/s/file?id=ba62/PLOSOne_formatting_sample_title_authors_affiliations.pdf.

2. Thank you for stating in your Funding Statement:

“Research reported in this publication was supported by the Department of Veterans Affairs Health Systems Research (Dr Finlay, VA HSR IIR 20–040). Dr. Sox-Harris was funded by a grant from the Department of Veterans Affairs Health Systems Research (RCS 14-232).

The views expressed in this article are those of the authors and do not necessarily reflect the position or policy of the Department of Veterans Affairs or the United States government. The funding organization had no role in the study design; analysis and interpretation of data; in the writing of the report; or in the decision to submit the paper for publication.

Funder URL: https://www.hsrd.research.va.gov/”

Please provide an amended statement that declares *all* the funding or sources of support (whether external or internal to your organization) received during this study, as detailed online in our guide for authors at http://journals.plos.org/plosone/s/submit-now.  Please also include the statement “There was no additional external funding received for this study.” in your updated Funding Statement.

Please include your amended Funding Statement within your cover letter. We will change the online submission form on your behalf.

3. In the online submission form, you indicated that your data is available only on request from a third party. Please note that your Data Availability Statement is currently missing [the name of the third party contact or institution / contact details for the third party, such as an email address or a link to where data requests can be made]. Please update your statement with the missing information.

4. If the reviewer comments include a recommendation to cite specific previously published works, please review and evaluate these publications to determine whether they are relevant and should be cited. There is no requirement to cite these works unless the editor has indicated otherwise.

[Note: HTML markup is below. Please do not edit.]

Reviewers' comments:

Reviewer's Responses to Questions

**Comments to the Author**

1. Is the manuscript technically sound, and do the data support the conclusions?

Reviewer #1: Yes

Reviewer #2: Yes

Reviewer #3: Partly

**********

2. Has the statistical analysis been performed appropriately and rigorously?

Reviewer #1: Yes

Reviewer #2: Yes

Reviewer #3: Yes

**********

3. Have the authors made all data underlying the findings in their manuscript fully available?

The PLOS Data policy

Reviewer #1: No

Reviewer #2: No

Reviewer #3: No

**********

4. Is the manuscript presented in an intelligible fashion and written in standard English?

Reviewer #1: Yes

Reviewer #2: Yes

Reviewer #3: Yes

**********

Reviewer #1: This manuscript describes methodology for accurately capturing Veterans eligible for care through VHA that have criminal legal involvement. The authors note that three administrative/clinical databases may capture this legal involvement, but for various reasons they are each incomplete. Identifying the "completeness" of each database compared with the overall merged dataset (there is no "gold standard" for comparison) helps standardize populations for studies--improving overall quality, reliability, and reproducibility.

Reviewer #2: Thank you for the opportunity to review this manuscript is best read as a methodological, descriptive study (not a traditional narrative “review”) that examines how to identify Veterans Health Administration (VHA) patients with contact with Veterans Justice Programs (VJP) using routinely available electronic health record/administrative data.

The topic is important, and the paper highlights a real problem: different data sources identify different groups of veterans, which can affect research and quality improvement efforts.

Several important issues should be noted.

Major comments:

1. Without a reference standard, the work cannot quantify sensitivity/specificity or false positives, so the core conclusion (“use all three”) is reasonable but not definitively proven as “most accurate.” This limits scientific impact because it remains unclear how close the combined method is to “truth.”

2. The study convincingly shows disagreement across sources, but does not identify which veterans are missed by each method or whether missedness is patterned by site, setting (court vs jail), or program workflow—issues that directly affect downstream bias in etiologic or quality-of-care studies.

3. Because documentation and EHR environments evolve, a one-year analysis may not generalize across time or modernization phases; this matters if the paper’s goal is to offer a durable standard definition for ongoing research.

4. Excluding veterans with VJP contact who are ineligible for VHA care narrows the population and may obscure important policy-relevant groups (e.g., those falling through coverage gaps), depending on the intended use of the algorithm.

5.The paper’s direct applicability is largely confined to VHA/VJP; for journals with broader readership, this reduces perceived contribution unless paired with a stronger methodological template transferable to other systems.

I recommend major revision, and I encourage you to continue this important line of work.

Reviewer #3: Dear Authors,

I have reviewed your manuscript, “Assessing Methods to Identify Veteran Health Administration Populations with Veterans Justice Programs Contact to Improve Health Research.” Overall, this is a practically important methods paper for VHA researchers and operational teams. The core idea, demonstrating that reliance on a single data source materially under-identifies VJP contact, is compelling and well aligned with PLOS ONE’s emphasis on methodological rigor and reproducibility. However, I recommend major revisions before the work is ready for publication, mainly to (i) resolve internal inconsistencies in counts, (ii) strengthen reproducibility of the cohort-identification algorithm, and (iii) address PLOS ONE expectations for transparent “data underlying the results” given restricted VA data.

Abstract

1) Fix all sample-size inconsistencies and align with the cohort flow (Fig 1) and Results text. The abstract reports 52,548 VHA-eligible veterans, while the Results/flow indicate Any VJP contact N=53,617 and Ineligible excluded N=950, which do not reconcile arithmetically. Ensure the same numbers appear in Abstract, Results, and figures, and explicitly state any additional exclusions if applicable.

2) Define the unit of “contact” and “identified through.” Clarify whether a “contact” is an encounter, a note/form record, a unique day, or some other operational unit, especially since you report “average number of 5.3 contacts (range 1–120)” without defining the metric.

Introduction

3) Clearly distinguish “criminal legal involvement” from “VJP contact.” Frame the primary outcome as VJP contact (an ascertainable EHR construct) and acknowledge that it is an imperfect proxy for broader criminal legal involvement. This prevents over-claiming and matches what the data actually means.

4) Strengthen the rationale for the three-source approach with expected bias mechanisms. You already explain why HOMES may be incomplete; add parallel expectations for: Stop codes (coding variability, missed/incorrect workload documentation); Note titles (facility-specific naming conventions, EHR modernization effects)

5) Consider tighten study aims into 1–2 explicit objectives. For example: (1) quantify overlap and under-ascertainment when using single vs multi-source definitions; (2) provide a reproducible cohort-identification specification usable in CDW/VINCI contexts.

Methodology

6) Make the identification algorithm reproducible (publishable specification). For each source, report: exact CDW tables/fields, inclusion filters, date windows, and linkage keys; define deduplication rules across sources.

7) Stop codes: specify data origin and operationalization. State which workload/encounter tables were queried, how stop codes were applied, and whether multiple encounters per person were counted (and how).

8) HOMES: specify the exact “entry/exit” definition and data handling. Identify the forms/fields used, how multiple records were handled, and how veterans were classified when entry/exit dates were missing.

9) Define “contact” operationally and specify how you computed “average contacts.” If combining heterogeneous records (encounters + forms + notes), define whether “contacts” are raw records, unique days, or events.

10) Clarify EHR modernization considerations. If you list Millennium sites, state whether you analyzed differences by system (even descriptively). If not, specify this clearly as a limitation and discuss likely direction of bias.

11) ICD-10 comparison: correct code inconsistencies and define the time window. Table 1 uses Y92.14x, while the Results text refers to “Y92.4” (likely a typo). Also clarify whether ICD-10 codes were searched only in FY2024 or historically.

12) Data Availability (PLOS ONE): provide a concrete access pathway + share underlying aggregates and code lists. Even if individual-level VA data cannot be public, provide: how qualified researchers can request access (governance route), and aggregated counts underlying tables/figures + full code lists/queries as Supporting Information.

Results

13) Reconcile all denominators across text, tables, and figures. The “Any VJP contact / ineligible excluded / eligible” counts currently do not reconcile. The HCRV sample size differs between text/Table 2 (n=8.541) and Fig 3a (n=8.556). Fix these before resubmission; inconsistent denominators are a major credibility issue.

14) Add a simple table quantifying “single-source capture” and “two-source capture.” Venn diagrams are helpful, but readers need a clear numeric summary of how many veterans would be missed if using: stop codes only; HOMES only; note titles only; each pairwise combination. This directly supports your core recommendation (“use all three sources”).

15) Ensure subgroup reporting (HCRV and VJO) is consistent with figures. Verify overlap percentages and Ns for HCRV/VJO across all outputs; align text statements with Fig 3a/3b.

Discussion

16) Temper over-strong claims about imputation and missing data. Instead of asserting imputation would be “unreliable,” specify that high missingness and likely non-random mechanisms may limit correction; emphasize that HOMES alone is insufficient without multi-source augmentation.

17) Provide practical guardrails to minimize stigmatization risk. You mention discrimination concerns; add 1–2 concrete implementation principles (role-based access, purpose limitation) to align operational use with ethical practice.

18) Briefly reinforce transparency commitments. State that code lists and aggregate data underlying figures/tables are provided in Supplementary files (once added), consistent with PLOS ONE transparency expectations.

Conclussion

19) Make the conclusion explicitly methodological and avoid overgeneralizing to “criminal legal involvement.” Use “veterans with VJP contact” as the measured construct.

20) State the actionable recommendation and the deliverable. Example structure: Single-source ascertainment materially under-identifies VJP contact. A standardized multi-source definition is recommended.

Overall

Your manuscript addresses an important VHA methods problem, but it needs key fixes before it can be re-evaluated: (1) reconcile all sample-size/denominator inconsistencies across the Abstract, Results, and Fig 1; (2) define the operational unit of “contact” and “identified through” (and how contacts are counted/merged across stop codes, HOMES, and notes); and (3) consistently frame the primary outcome as “VJP contact” (an EHR-ascertainable construct) rather than broader “criminal legal involvement,” which is only a proxy. Therefore I recocommended major revision.

**********

what does this mean?). If published, this will include your full peer review and any attached files.

If you choose “no”, your identity will remain anonymous but your review may still be made public.

**Do you want your identity to be public for this peer review?**  For information about this choice, including consent withdrawal, please see our Privacy Policy

Reviewer #1: **Yes:**  Dennis Faix

Reviewer #2: **Yes:**  Ming Zheng

Reviewer #3: No

**********

[NOTE: If reviewer comments were submitted as an attachment file, they will be attached to this email and accessible via the submission site. Please log into your account, locate the manuscript record, and check for the action link "View Attachments". If this link does not appear, there are no attachment files.]

To ensure your figures meet our technical requirements, please review our figure guidelines: https://journals.plos.org/plosone/s/figures

You may also use PLOS’s free figure tool, NAAS, to help you prepare publication quality figures: https://journals.plos.org/plosone/s/figures#loc-tools-for-figure-preparation.

NAAS will assess whether your figures meet our technical requirements by comparing each figure against our figure specifications.

# Author comment: Author response to Decision Letter 1

**DOI:** 10.1371/journal.pone.0357665.r002

7 Jul 2026

Dear Dr. Cioffi,

We thank you and the reviewers for the thoughtful comments on our manuscript entitled, “Assessing methods to identify Veteran Health Administration populations with Veterans Justice Programs contact to improve health research” (PONE-D-25-62496). We greatly appreciate your insightful feedback and have no doubt our manuscript is strengthened as a result. Below are the comments followed by our responses.

Editor

Comment #1: Thank you for submitting your manuscript to PLOS ONE. After careful consideration, we feel that it has merit but does not fully meet PLOS ONE’s publication criteria as it currently stands. Therefore, we invite you to submit a revised version of the manuscript that addresses the points raised during the review process.

Response: Thank you!

Comment #2: The most significant issue concerns the methodological validation of the proposed identification strategy. The manuscript convincingly demonstrates that reliance on any single data source would lead to incomplete ascertainment of veterans with Veterans Justice Programs (VJP) contact. However, the current analysis remains largely descriptive and does not provide a formal validation of the combined approach. In the absence of a gold standard, the recommendation to use all three data sources is reasonable but presently insufficiently substantiated. The manuscript would be strengthened by additional analyses that assess concordance between sources, quantify agreement, and estimate potential misclassification. Where feasible, validation using chart review, linkage to external justice system data, or sensitivity analyses exploring alternative definitions would substantially improve the methodological rigor. If a gold standard is not available, this limitation should be more fully explored and the conclusions framed more cautiously.

Response: To assess concordance between sources, we included Fleiss’ kappa tests for VJP, HCRV, and VJO. To examine validation, we tested patient and facility factors associated with each type of data source using the overall VJP sample. Results indicated that patient factors varied in their association with each type of data source. For example, female veterans had lower odds of having a HOMES records and higher odds of having a chart note compared to male veterans. Given that there is no gold standard available, we expanded our limitations to more fully explore this point and framed our conclusions more cautiously.

Comment #3: A related concern is the limited statistical depth of the analysis. The current presentation focuses primarily on counts, percentages, and descriptive overlap across data sources. For a methodological study, readers would benefit from more formal measures of agreement and reliability, such as kappa statistics or other concordance metrics, as well as sensitivity analyses examining how population characteristics or program-level factors influence identification across sources. Additional analyses clarifying the implications of using one versus multiple sources for downstream research would also enhance the contribution of the paper.

Response: Thank you for these suggestions. As described in our response to Comment #2, we added Fleiss’ kappa tests, examined the association between patient and facility factors and different data sources. We also explored variation in the prevalence of opioid use disorder diagnosis and receipt of medications for opioid use disorder depending on the data source used. We also calculated the prevalence of a clinical condition (i.e., opioid use disorder) and a quality measure for this clinical condition (i.e., receipt of medications for opioid use disorder). Depending on the data source used, the prevalence of opioid use disorder ranged from 5.3% for clinic code only group to 13.8% for the all three data sources group (an 8.5% range). Receipt of medications for opioid use disorder ranged from 51.3% for the clinic codes + HOMES group to 57.1% for the HOMES + chart note group (a 5.8% range). Clinic conditions and performance measures are monitored at the VHA; thus, it is important to use the most accurate approach to identifying the population of interest to ensure that other measures based on this population sample are correctly calculated and reported.

Comment #4: The discussion section would also benefit from further refinement. At present, the conclusions occasionally extend beyond what can be supported by the data, particularly in recommending the universal use of all three data sources. These recommendations should be framed more cautiously and clearly situated within the limitations of the study design. A more explicit discussion of generalizability—both within the VHA and in relation to other healthcare systems—would also strengthen the manuscript.

Response: We extensively revised the Discussion section to frame our results more cautiously and describe the generalizability of the results.

Comment #5: Finally, several technical and reporting issues should be addressed in revision. The data availability statement should be clarified to ensure full compliance with journal policy.

Response: We have updated the data availability statement.

Comment #6: Some sections of the discussion repeat points made earlier and could be streamlined for clarity.

Response: We revised and streamlined the discussion section.

Comment #7: Figures illustrating overlap across data sources are useful but would benefit from additional interpretation within the text.

Response: Additional information on how to interpret the figure was added to the Results.

Comment #8: The exploratory analysis of ICD-10 codes is interesting but currently underdeveloped; either expanding its analytic contribution or more clearly framing it as preliminary would improve coherence.

Response: Given that we added new analyses, we decided to remove the exploratory ICD-10 codes and focus our analyses on the data sources.

Comment #9: In summary, we believe this manuscript addresses an important methodological challenge and has the potential to make a meaningful contribution. However, substantial revision is required to strengthen the validation strategy, expand the analytic depth, and align the conclusions more closely with the evidence presented. We would welcome a carefully revised version that addresses these and reviewers' concerns.

Response: Thank you – we look forward to seeing your comments on our revised manuscript.

Comment #10: When submitting your revision, we need you to address these additional requirements.

1. Please ensure that your manuscript meets PLOS ONE's style requirements, including those for file naming. The PLOS ONE style templates can be found at

https://journals.plos.org/plosone/s/file?id=wjVg/PLOSOne_formatting_sample_main_body.pdf and

https://journals.plos.org/plosone/s/file?id=ba62/PLOSOne_formatting_sample_title_authors_affiliations.pdf.

Response: We aligned and double checked our revisions to meet PLOS ONE’s style requirements.

Comment #11: 2. Thank you for stating in your Funding Statement:

“Research reported in this publication was supported by the Department of Veterans Affairs Health Systems Research (Dr Finlay, VA HSR IIR 20–040). Dr. Sox-Harris was funded by a grant from the Department of Veterans Affairs Health Systems Research (RCS 14-232).

The views expressed in this article are those of the authors and do not necessarily reflect the position or policy of the Department of Veterans Affairs or the United States government. The funding organization had no role in the study design; analysis and interpretation of data; in the writing of the report; or in the decision to submit the paper for publication.

Funder URL: https://www.hsrd.research.va.gov/”

Please provide an amended statement that declares *all* the funding or sources of support (whether external or internal to your organization) received during this study, as detailed online in our guide for authors at http://journals.plos.org/plosone/s/submit-now. Please also include the statement “There was no additional external funding received for this study.” in your updated Funding Statement.

Please include your amended Funding Statement within your cover letter. We will change the online submission form on your behalf.

Response: The amended Funding Statement is included in the cover letter.

Comment #12: 3. In the online submission form, you indicated that your data is available only on request from a third party. Please note that your Data Availability Statement is currently missing [the name of the third party contact or institution / contact details for the third party, such as an email address or a link to where data requests can be made]. Please update your statement with the missing information.

Response: The Data Availability Statement has been revised to include this information.

Comment #13: 4. If the reviewer comments include a recommendation to cite specific previously published works, please review and evaluate these publications to determine whether they are relevant and should be cited. There is no requirement to cite these works unless the editor has indicated otherwise.

Response: No specific previously published works were recommended by the reviewers.

Reviewer #1

This manuscript describes methodology for accurately capturing Veterans eligible for care through VHA that have criminal legal involvement. The authors note that three administrative/clinical databases may capture this legal involvement, but for various reasons they are each incomplete. Identifying the "completeness" of each database compared with the overall merged dataset (there is no "gold standard" for comparison) helps standardize populations for studies--improving overall quality, reliability, and reproducibility.

Response: Thank you!

Reviewer #2

Thank you for the opportunity to review this manuscript is best read as a methodological, descriptive study (not a traditional narrative “review”) that examines how to identify Veterans Health Administration (VHA) patients with contact with Veterans Justice Programs (VJP) using routinely available electronic health record/administrative data.

The topic is important, and the paper highlights a real problem: different data sources identify different groups of veterans, which can affect research and quality improvement efforts.

Response: Thank you!

Several important issues should be noted.

Major comments:

Comment #1: Without a reference standard, the work cannot quantify sensitivity/specificity or false positives, so the core conclusion (“use all three”) is reasonable but not definitively proven as “most accurate.” This limits scientific impact because it remains unclear how close the combined method is to “truth.”

Response: We have expanded upon this limitation and noted that it may not be feasible to compared these data with data from criminal legal sources because of the number of potential sources (i.e., law enforcement for arrests, courts, jails, prisons, and community supervision departments). We have also tempered our conclusions to reflect this limitation.

Comment #2: The study convincingly shows disagreement across sources, but does not identify which veterans are missed by each method or whether missedness is patterned by site, setting (court vs jail), or program workflow—issues that directly affect downstream bias in etiologic or quality-of-care studies.

Response: We added additional analyses to examine the association between patient and facility factors and different data sources, and explored variation in the prevalence of opioid use disorder diagnosis and receipt of medications for opioid use disorder depending on the data source used to shed light on differences that would be observed depending on the data sources used.

Comment #3: Because documentation and EHR environments evolve, a one-year analysis may not generalize across time or modernization phases; this matters if the paper’s goal is to offer a durable standard definition for ongoing research.

Response: We included two additional Fiscal Years of data, 2014 and 2019, and examined changes in data sources over time.

Comment #4: Excluding veterans with VJP contact who are ineligible for VHA care narrows the population and may obscure important policy-relevant groups (e.g., those falling through coverage gaps), depending on the intended use of the algorithm.

Response: We now note in the Methods section that we excluded 929 veterans in Fiscal Year 2024 because they were ineligible for VHA care or who did not have CDW electronic health records because these veterans did not have the patient information necessary for our exploration of patient factors associated with different data sources and examination of diagnosis and receipt of medications for opioid use disorder. We also added this limitation to Discussion section.

Comment #5: The paper’s direct applicability is largely confined to VHA/VJP; for journals with broader readership, this reduces perceived contribution unless paired with a stronger methodological template transferable to other systems.

Response: We agree that the direct applicability is largely focused on VHA and have added this as a limitation.

Comment #6: I recommend major revision, and I encourage you to continue this important line of work.

Response: Thank you!

Reviewer #3:

I have reviewed your manuscript, “Assessing Methods to Identify Veteran Health Administration Populations with Veterans Justice Programs Contact to Improve Health Research.” Overall, this is a practically important methods paper for VHA researchers and operational teams. The core idea, demonstrating that reliance on a single data source materially under-identifies VJP contact, is compelling and well aligned with PLOS ONE’s emphasis on methodological rigor and reproducibility. However, I recommend major revisions before the work is ready for publication, mainly to (i) resolve internal inconsistencies in counts, (ii) strengthen reproducibility of the cohort-identification algorithm, and (iii) address PLOS ONE expectations for transparent “data underlying the results” given restricted VA data.

Response: Thank you – we have resolved internal inconsistencies, strengthened the reproducibility of the cohort-identification algorithm, and provided additional information about data access. Details are described below. For example, we now include S1 Appendix with specifications for the algorithm. We also updated the Data Availability Statement to include contact information to request data access.

Abstract

Comment #1: Fix all sample-size inconsistencies and align with the cohort flow (Fig 1) and Results text. The abstract reports 52,548 VHA-eligible veterans, while the Results/flow indicate Any VJP contact N=53,617 and Ineligible excluded N=950, which do not reconcile arithmetically. Ensure the same numbers appear in Abstract, Results, and figures, and explicitly state any additional exclusions if applicable.

Response: We triple-checked all numbers to ensure they align throughout the documents.

Comment #2: Define the unit of “contact” and “identified through.” Clarify whether a “contact” is an encounter, a note/form record, a unique day, or some other operational unit, especially since you report “average number of 5.3 contacts (range 1–120)” without defining the metric.

Response: We added the definition of contact. We removed the term “identified through” to improve clarity. We removed the average contacts from the Results section for simplicity as our manuscript is not focused on multiple contacts, but rather on identifying veterans who have any contact with VJP.

Introduction

Comment #3: Clearly distinguish “criminal legal involvement” from “VJP contact.” Frame the primary outcome as VJP contact (an ascertainable EHR construct) and acknowledge that it is an imperfect proxy for broader criminal legal involvement. This prevents over-claiming and matches what the data actually means.

Response: We revised throughout the manuscript to clarify that we were identifying VJP contact and that it is an imperfect proxy for broader criminal legal involvement.

Comment #4: Strengthen the rationale for the three-source approach with expected bias mecha

Attachment Submitted filename: Response_to_reviewers.docx

# Peer review: Decision Letter 1

**DOI:** 10.1371/journal.pone.0357665.r003

**Authors:** Andrea Cioffi

15 Jul 2026

Dear Dr. Finlay,

Thank you for submitting your manuscript to PLOS ONE. After careful consideration, we feel that it has merit but does not fully meet PLOS ONE’s publication criteria as it currently stands. Therefore, we invite you to submit a revised version of the manuscript that addresses the points raised during the review process.

Please, see Additional Editor Comments and Reviewer's comments below.

Please submit your revised manuscript by Aug 29 2026 11:59PM. If you will need more time than this to complete your revisions, please reply to this message or contact the journal office at plosone@plos.org. When you're ready to submit your revision, log on to https://www.editorialmanager.com/pone/ and select the 'Submissions Needing Revision' folder to locate your manuscript file.

- A letter that responds to each point raised by the academic editor and reviewer(s). You should upload this letter as a separate file labeled 'Response to Reviewers'.
- A marked-up copy of your manuscript that highlights changes made to the original version. You should upload this as a separate file labeled 'Revised Manuscript with Track Changes'.
- An unmarked version of your revised paper without tracked changes. You should upload this as a separate file labeled 'Manuscript'.

If you would like to make changes to your financial disclosure, please include your updated statement in your cover letter. Guidelines for resubmitting your figure files are available below the reviewer comments at the end of this letter.

If applicable, we recommend that you deposit your laboratory protocols in protocols.io to enhance the reproducibility of your results. Protocols.io assigns your protocol its own identifier (DOI) so that it can be cited independently in the future. For instructions see: https://journals.plos.org/plosone/s/submission-guidelines#loc-laboratory-protocols. Additionally, PLOS ONE offers an option for publishing peer-reviewed Lab Protocol articles, which describe protocols hosted on protocols.io. Read more information on sharing protocols at https://plos.org/protocols?utm_medium=editorial-email&utm_source=authorletters&utm_campaign=protocols.

As the corresponding author, your ORCID iD is verified in the submission system and will appear in the published article. PLOS supports the use of ORCID, and we encourage all coauthors to register for an ORCID iD and use it as well. Please encourage your coauthors to verify their ORCID iD within the submission system before final acceptance, as unverified ORCID iDs will not appear in the published article. *Only*  the individual author can complete the verification step; PLOS staff *cannot*  verify ORCID iDs on behalf of authors.

We look forward to receiving your revised manuscript.

Kind regards,

Andrea Cioffi

Academic Editor

PLOS One

Journal Requirements:

If the reviewer comments include a recommendation to cite specific previously published works, please review and evaluate these publications to determine whether they are relevant and should be cited. There is no requirement to cite these works unless the editor has indicated otherwise.

Please review your reference list to ensure that it is complete and correct. If you have cited papers that have been retracted, please include the rationale for doing so in the manuscript text, or remove these references and replace them with relevant current references. Any changes to the reference list should be mentioned in the rebuttal letter that accompanies your revised manuscript. If you need to cite a retracted article, indicate the article’s retracted status in the References list and also include a citation and full reference for the retraction notice.

Additional Editor Comments:

Although the manuscript is now close to being suitable for publication, I believe that a few final revisions would further strengthen the presentation.

First, while the conclusions have been moderated appropriately compared with the previous version, I encourage the authors to maintain consistently cautious language when recommending the combined use of all three data sources. Given the absence of an external reference standard, it cannot be definitively established that the combined approach represents the most accurate method of identifying veterans with VJP contact. Rather, the evidence supports the conclusion that, among the approaches evaluated in this study, the combined strategy provides the most comprehensive ascertainment. Maintaining this distinction throughout the Discussion and Conclusions will ensure that the recommendations remain fully aligned with the available evidence.

Second, although the limitations section has been substantially expanded, a brief additional emphasis on the implications of the absence of a true gold standard would be valuable. Specifically, it would be helpful to reiterate that the study evaluates concordance among existing data sources rather than diagnostic accuracy, and that future validation against external justice-system data, where feasible, would further strengthen confidence in the proposed identification strategy.

Finally, I encourage the authors to perform one final careful review of the manuscript to ensure complete consistency of terminology, numerical values, and descriptions across the Abstract, Results, tables, figures, and Supporting Information. The revised manuscript appears considerably improved in this regard, and a final editorial check will help ensure complete consistency throughout.

[Note: HTML markup is below. Please do not edit.]

Reviewers' comments:

Reviewer's Responses to Questions

**Comments to the Author**

Reviewer #2: All comments have been addressed

Reviewer #3: All comments have been addressed

**********

2. Is the manuscript technically sound, and do the data support the conclusions?

Reviewer #2: Yes

Reviewer #3: Partly

**********

3. Has the statistical analysis been performed appropriately and rigorously?

Reviewer #2: Yes

Reviewer #3: Yes

**********

4. Have the authors made all data underlying the findings in their manuscript fully available?

The PLOS Data policy

Reviewer #2: Yes

Reviewer #3: Yes

**********

5. Is the manuscript presented in an intelligible fashion and written in standard English?

Reviewer #2: Yes

Reviewer #3: Yes

**********

Reviewer #2: Thank you for providing the revised manuscript and responses to the comments. I have reviewed the revision and have no further questions or comments.

Reviewer #3: Dear Authors,

Thank you for the revised version of the manuscript. The manuscript has been substantially improved, and the main methodological concerns raised in the previous review have largely been addressed. In particular, the cohort counts are now much clearer, the primary construct is better framed as VJP contact, the operational definition of contact has been added, and Table 2 provides a useful summary of single-source and combined-source identification patterns.

I recommend only minor revisions before publication.

Please address the following remaining points:

1. Align percentages across the Abstract, Results, and tables.

The Abstract reports 81% clinic stop codes and 64% chart note titles, whereas the Results section/ under Table 2 (row 230 and 231), reports 80% and 63%, respectively. Please harmonize these values throughout the manuscript.

2. Further refine the framing of VJP contact vs. criminal legal involvement.

The manuscript is now much clearer, but one sentence in the Introduction still states that the focus is on veterans with criminal legal involvement (row 82-86). Please consider revising this to emphasize that the study focuses on veterans with VJP contact, used as a proxy for broader criminal legal involvement.

3. Correct minor typographical and abbreviation issues.

For example, Table 2 includes “Fiscsl Year 2024,” and the table note appears to define VJP twice, including one instance where “VJP = Veterans Justice Outreach” should likely be “VJO = Veterans Justice Outreach.”

Overall, the revised manuscript is much stronger and now provides a clearer and more useful methodological contribution. After these minor corrections, I believe the manuscript will be suitable for publication.

**********

what does this mean?). If published, this will include your full peer review and any attached files.

If you choose “no”, your identity will remain anonymous but your review may still be made public.

**Do you want your identity to be public for this peer review?**  For information about this choice, including consent withdrawal, please see our Privacy Policy

Reviewer #2: No

Reviewer #3: **Yes:**  Michal Vagner

**********

[NOTE: If reviewer comments were submitted as an attachment file, they will be attached to this email and accessible via the submission site. Please log into your account, locate the manuscript record, and check for the action link "View Attachments". If this link does not appear, there are no attachment files.]

To ensure your figures meet our technical requirements, please review our figure guidelines: https://journals.plos.org/plosone/s/figures

You may also use PLOS’s free figure tool, NAAS, to help you prepare publication quality figures: https://journals.plos.org/plosone/s/figures#loc-tools-for-figure-preparation.

NAAS will assess whether your figures meet our technical requirements by comparing each figure against our figure specifications.

# Author comment: Author response to Decision Letter 2

**DOI:** 10.1371/journal.pone.0357665.r004

17 Aug 2026

Dear Dr. Cioffi,

We thank you and the reviewers for the thoughtful comments on our revised manuscript entitled, “Assessing methods to identify Veteran Health Administration populations with Veterans Justice Programs contact to improve health research” (PONE-D-25-62496R1). We greatly appreciate your insightful feedback and have no doubt our manuscript is strengthened as a result. Below are the comments followed by our responses.

Editor

Journal Requirements:

Comment #1: If the reviewer comments include a recommendation to cite specific previously published works, please review and evaluate these publications to determine whether they are relevant and should be cited. There is no requirement to cite these works unless the editor has indicated otherwise.

Response: There were no recommendations from reviewers to cite specific previously published works.

Comment #2: Please review your reference list to ensure that it is complete and correct. If you have cited papers that have been retracted, please include the rationale for doing so in the manuscript text, or remove these references and replace them with relevant current references. Any changes to the reference list should be mentioned in the rebuttal letter that accompanies your revised manuscript. If you need to cite a retracted article, indicate the article’s retracted status in the References list and also include a citation and full reference for the retraction notice.

Response: We reviewed the reference list to ensure it was complete and did not identify any cited papers that were retracted. We updated one reference, replacing the Deyo comorbidity index with an updated version of the Charlson comorbidity index from 2005. We also made minor modifications to some references (e.g., changing a full journal name to the journal abbreviation).

Additional Editor Comments:

Comment #3: Although the manuscript is now close to being suitable for publication, I believe that a few final revisions would further strengthen the presentation. First, while the conclusions have been moderated appropriately compared with the previous version, I encourage the authors to maintain consistently cautious language when recommending the combined use of all three data sources. Given the absence of an external reference standard, it cannot be definitively established that the combined approach represents the most accurate method of identifying veterans with VJP contact. Rather, the evidence supports the conclusion that, among the approaches evaluated in this study, the combined strategy provides the most comprehensive ascertainment. Maintaining this distinction throughout the Discussion and Conclusions will ensure that the recommendations remain fully aligned with the available evidence.

Response: We updated the Abstract, Discussion, and Conclusions to temper our recommendations for the use of all three data sources to ensure we are aligned with the available evidence.

Comment #4: Second, although the limitations section has been substantially expanded, a brief additional emphasis on the implications of the absence of a true gold standard would be valuable. Specifically, it would be helpful to reiterate that the study evaluates concordance among existing data sources rather than diagnostic accuracy, and that future validation against external justice-system data, where feasible, would further strengthen confidence in the proposed identification strategy.

Response: We have added this additional emphasis to the Limitations section to reiterate the focus on evaluating concordance among existing data sources rather than diagnostic accuracy. We also revised the following sentence “future research…” to “future validation against external criminal legal system data, where feasible, would further strengthen confidence in the proposed identification strategy.”

Comment #5: Finally, I encourage the authors to perform one final careful review of the manuscript to ensure complete consistency of terminology, numerical values, and descriptions across the Abstract, Results, tables, figures, and Supporting Information. The revised manuscript appears considerably improved in this regard, and a final editorial check will help ensure complete consistency throughout.

Response: We had two authors conduct a complete check of the manuscript and appendices to ensure consistency throughout.

Reviewer #2

Initial Comment: Thank you for providing the revised manuscript and responses to the comments. I have reviewed the revision and have no further questions or comments.

Response: Thank you!

Reviewer #3

Initial Comment: Thank you for the revised version of the manuscript. The manuscript has been substantially improved, and the main methodological concerns raised in the previous review have largely been addressed. In particular, the cohort counts are now much clearer, the primary construct is better framed as VJP contact, the operational definition of contact has been added, and Table 2 provides a useful summary of single-source and combined-source identification patterns. I recommend only minor revisions before publication.

Response: Thank you!

Comment #1: Align percentages across the Abstract, Results, and tables. The Abstract reports 81% clinic stop codes and 64% chart note titles, whereas the Results section/ under Table 2 (row 230 and 231), reports 80% and 63%, respectively. Please harmonize these values throughout the manuscript.

Response: We had two authors conduct a complete check of the manuscript and appendices to ensure consistency throughout and harmonized any mismatched values.

Comment #2: Further refine the framing of VJP contact vs. criminal legal involvement. The manuscript is now much clearer, but one sentence in the Introduction still states that the focus is on veterans with criminal legal involvement (row 82-86). Please consider revising this to emphasize that the study focuses on veterans with VJP contact, used as a proxy for broader criminal legal involvement.

Response: We clarified in the Introduction that this study was limited to veterans who received VJP outreach services from HCRV and VJO staff. The following sentence now notes that researchers have used encounters with these programs as a broad proxy for criminal legal involvement.

Comment #3: Correct minor typographical and abbreviation issues. For example, Table 2 includes “Fiscsl Year 2024,” and the table note appears to define VJP twice, including one instance where “VJP = Veterans Justice Outreach” should likely be “VJO = Veterans Justice Outreach.” Overall, the revised manuscript is much stronger and now provides a clearer and more useful methodological contribution. After these minor corrections, I believe the manuscript will be suitable for publication.

Response: We corrected the minor typographical and abbreviations noted, including changing “Fiscsl” to “Fiscal” and “VJP” to “VJO” in Table 2. We also had two authors check the manuscript closely for any additional typos.

We appreciate the opportunity to revise our manuscript.

Thank you,

The Authors

Attachment Submitted filename: Response to Reviewers.docx

# Editor report: Decision Letter 2

**DOI:** 10.1371/journal.pone.0357665.r005

**Authors:** Andrea Cioffi

20 Aug 2026

Assessing methods to identify Veterans Health Administration populations with Veterans Justice Programs contact to improve health research

PONE-D-25-62496R2

Dear Dr. Finlay,

We’re pleased to inform you that your manuscript has been judged scientifically suitable for publication and will be formally accepted for publication once it meets all outstanding technical requirements.

Within one week, you’ll receive an e-mail detailing the required amendments. When these have been addressed, you’ll receive a formal acceptance letter and your manuscript will be scheduled for publication.

An invoice will be generated when your article is formally accepted. Please note, if your institution has a publishing partnership with PLOS and your article meets the relevant criteria, all or part of your publication costs will be covered. Please make sure your user information is up-to-date by logging into Editorial Manager at Editorial Manager® and clicking the ‘Update My Information' link at the top of the page. For questions related to billing, please contact billing support.

If your institution or institutions have a press office, please notify them about your upcoming paper to help maximize its impact. If they’ll be preparing press materials, please inform our press team as soon as possible -- no later than 48 hours after receiving the formal acceptance. Your manuscript will remain under strict press embargo until 2 pm Eastern Time on the date of publication. For more information, please contact onepress@plos.org.

Kind regards,

Andrea Cioffi

Academic Editor

PLOS One

Additional Editor Comments (optional):

Reviewers' comments:

# Editor report: Acceptance letter

**DOI:** 10.1371/journal.pone.0357665.r006

**Authors:** Andrea Cioffi

PONE-D-25-62496R2

PLOS One

Dear Dr. Finlay,

I'm pleased to inform you that your manuscript has been deemed suitable for publication in PLOS One. Congratulations! Your manuscript is now being handed over to our production team.

At this stage, our production department will prepare your paper for publication. This includes ensuring the following:

* All references, tables, and figures are properly cited

* All relevant supporting information is included in the manuscript submission,

* There are no issues that prevent the paper from being properly typeset

You will receive further instructions from the production team, including instructions on how to review your proof when it is ready. Please keep in mind that we are working through a large volume of accepted articles, so please give us a few days to review your paper and let you know the next and final steps.

Lastly, if your institution or institutions have a press office, please let them know about your upcoming paper now to help maximize its impact. If they'll be preparing press materials, please inform our press team within the next 48 hours. Your manuscript will remain under strict press embargo until 2 pm Eastern Time on the date of publication. For more information, please contact onepress@plos.org.

You will receive an invoice from PLOS for your publication fee after your manuscript has reached the completed accept phase. If you receive an email requesting payment before acceptance or for any other service, this may be a phishing scheme. Learn how to identify phishing emails and protect your accounts at https://explore.plos.org/phishing.

If we can help with anything else, please email us at customercare@plos.org.

Thank you for submitting your work to PLOS One and supporting open access.

Kind regards,

PLOS One Editorial Office Staff

on behalf of

Dr. Andrea Cioffi

Academic Editor

PLOS One
